# Understanding the Secular Decline in Testosterone: Mechanisms, Consequences, and Clinical Perspectives

**DOI:** 10.3390/ijms27020692

**Published:** 2026-01-09

**Authors:** Óscar Fraile-Martínez, Miguel A. Ortega, Cielo García-Montero

**Affiliations:** 1 Department of Medicine and Medical Specialities, Faculty of Medicine and Health Sciences, University of Alcala, 28801 Alcala de Henares, Spain; cielo.gmontero@gmail.com; 2 Ramón y Cajal Institute of Sanitary Research (IRYCIS), 28034 Madrid, Spain

**Keywords:** testosterone, secular decline, hypothalamic–pituitary–gonadal (HPGn) axis, lifestyle interventions, environmental exposure

## Abstract

Testosterone is a key regulator of male and female physiology, influencing reproductive function, muscle and bone anabolism, metabolic homeostasis, and psychological well-being. Growing evidence indicates a secular, age-independent decline in testosterone levels across populations, a trend associated with reduced fertility, metabolic and cardiovascular dysfunction, mood disturbances, and impaired quality of life. While aging and genetic factors play a role, a wide range of modifiable influences—including obesity, physical inactivity, unhealthy dietary patterns, chronic stress, poor sleep, and exposure to endocrine-disrupting chemicals or other environmental stressors—appear to contribute substantially to this phenomenon. This narrative review synthesizes the evidence on testosterone’s physiological significance, the causes and consequences of its secular decline, and evaluates potential interventions, emphasizing lifestyle and environmental strategies (physical activity, nutrition, weight management, sleep, stress reduction, sunlight exposure) as well as pharmacological and nutraceutical options. Overall, the contemporary testosterone decline represents a complex, multifactorial public health issue requiring integrated approaches to preserve hormonal and systemic health.

## 1. Introduction

Testosterone is one of the most extensively studied hormones in endocrinology, yet its clinical significance continues to evolve as our understanding of its complex physiological roles deepens. Since its discovery in the 1930s, testosterone has been at the center of discussions of male health, responsible for regulating sex differentiation, male phenotypic traits, fertility, and spermatogenesis [1]. More recently, testosterone has gained recognition for its contributions to overall well-being in both men and women due to its role in regulating various physiological processes, such as metabolic, bone, immune, and cardiovascular health, and its key influence on mental health and psychological functioning [2,3,4,5].

Testosterone levels are inherently dynamic, shaped by different factors such as age, genetics, lifestyle, environmental exposures, health status, and psychosocial modulators [6,7,8]. All these determinants and signals can directly or indirectly interact with the hypothalamic–pituitary–gonadal (HPGn) axis, which is responsible for orchestrating testosterone synthesis and levels [9]. While a gradual decline in testosterone with advancing age is well recognized—most notably in men aged 70–80 years, and progressively in women during the premenopausal period with a stabilization or a modest increase after menopause [10,11]—cumulative evidence has documented an additional, age-independent decline in men. In more detail, large-scale studies report reductions in serum testosterone of approximately 0.5–1.0% annually across diverse populations [12,13], indicative of a growing phenomenon with potential public health implications.

The causes of this intergenerational decline appear to be multifactorial and synergistic. Rising rates of obesity and metabolic disorders, exposure to endocrine-disrupting chemicals (EDCs) like phthalates or microplastics, sedentary lifestyles, unhealthy dietary patterns, and chronic psychosocial stress have all been implicated [14,15,16,17,18]. It seems that no single factor alone accounts for the observed reductions, suggesting a cumulative burden of metabolic, environmental, and behavioral stressors as the driving force.

The consequences of this testosterone deficiency are increasingly recognized in the scientific literature. From a medical perspective, low testosterone levels (clinically diagnosed as hypogonadism) are strongly associated with various chronic maladies including metabolic syndrome, cardiovascular disease and overall mortality [19,20,21,22,23]. Psychologically and socially, low testosterone levels are linked to increased rates of depression, anxiety, reduced motivation, lower quality of life, and impaired social behaviors such as competitiveness and engagement [24,25,26,27,28]. Additionally, emerging evidence suggests that lower testosterone levels may contribute to impaired sexual function, reduced fertility potential, and accelerated cognitive decline, with associations to dementia and neurodegenerative diseases [29,30,31]. These findings highlight that the secular decline in testosterone carries not only individual health risks but also broader demographic and societal implications.

Given the growing scope of this issue, various interventions are being explored to mitigate testosterone decline and its effects. Professional guidelines emphasize lifestyle-based strategies—such as weight loss, dietary changes, physical activity, sleep interventions and stress management—as first-line treatments [32,33,34,35]. When necessary, pharmacological approaches like testosterone replacement therapy (TRT) are available in multiple formulations, from injectables to transdermal gels and long-acting agents, reflecting ongoing efforts to balance efficacy with safety [32,36], along with certain herbal and nutritional supplements with proven scientific evidence [37,38].

In this review, we aim to synthesize current evidence on the relevance of testosterone both in men and women, focusing on the physiology of this hormone to better understand their multifaceted roles. Afterwards, the evidence, causes and consequences of the secular decline in men will be explored to conclude with both lifestyle and pharmacological strategies that may serve as effective interventions for this increasingly relevant global concern.

## 2. Search Strategy and Literature Selection

This manuscript is conceived as a narrative and integrative review rather than a systematic review. Accordingly, the objective was not to exhaustively capture all available publications, but to synthesize and critically interpret the most relevant and methodologically informative evidence addressing testosterone physiology, its secular decline, associated determinants, clinical consequences, and potential interventions.

A structured literature search was conducted using major biomedical databases, including PubMed/MEDLINE, Web of Science, Scopus, and Google Scholar, covering publications up to December 2025. Search terms were combined using Boolean operators and included, but were not limited to: “testosterone”, “androgens”, “secular decline”, “temporal trends”, “aging”, “hypogonadism”, “sex hormone–binding globulin”, “endocrine-disrupting chemicals”, “lifestyle factors”, “cardiometabolic risk”, and “testosterone replacement therapy”. Reference lists of key articles and relevant reviews were also manually screened to identify additional influential or foundational studies.

Given the broad and interdisciplinary scope of the review, the literature selection followed thematic relevance and scientific contribution rather than rigid temporal or methodological constraints. Priority was given to peer-reviewed original studies, large observational cohorts, meta-analyses, and authoritative reviews, with particular emphasis on publications from the last 10–15 years when addressing contemporary mechanisms, epidemiological trends, and clinical implications. However, seminal and historically important studies were included when necessary to contextualize testosterone physiology and the early evidence supporting secular trends.

Studies were preferentially selected based on methodological rigor, sample size, relevance to human physiology or clinical outcomes, and contribution to conceptual or mechanistic understanding. For the specific topic of secular testosterone decline, where the number of high-quality population-based studies is inherently limited, all major and frequently cited investigations were considered irrespective of publication year.

No formal exclusion criteria based on study design were applied, although case reports and non-peer-reviewed sources were generally excluded unless providing unique mechanistic or historical insights. Given the narrative nature of the review, formal risk-of-bias assessment and quantitative synthesis were not performed.

This methodological approach inherently carries limitations, including potential selection bias and reduced reproducibility compared with systematic reviews. Nevertheless, it allows a broader integrative and conceptual analysis, which is particularly valuable for complex, multifactorial phenomena such as the secular decline in testosterone.

## 3. An Overview of the Relevance of Testosterone: From Its Synthesis to Its Multifaceted Roles

### 3.1. Synthesis and Release of Testosterone

Testosterone is a steroid sex hormone belonging to the group of androgens. Androgens are classified as a 19-carbon steroid based on the androstane skeleton, being synthesized from cholesterol mainly in the testes (Leydig cells), ovaries, adrenal cortex, brain, and peripheral tissues like skin and fat [39]. Chemically, testosterone is a 17-beta-Hydroxy-4-Androsten-3-one. Other biologically relevant androgens include DHEA (dehydroepiandrosterone), A4 (4-androstenedione), DHT (dihydrotestosterone), KTs (11-keto-androgens), 17β-acyl-T (testosterone esters), androsterone (Ane), and androgenic pheromones (AP). These androgens are interconnected through dynamic metabolic pathways, where precursor molecules like DHEA and A4 are enzymatically converted into more active forms such as testosterone and DHT, or into alternative derivatives like 11-keto-testosterone (KT) or estrogenic androgens (EA), depending on the tissue, enzyme expression, and physiological context [39,40,41].

Testosterone synthesis is primarily regulated via the HPGn axis—although in males, as the synthesis mainly occurs in the testes, it is commonly referred as the hypothalamic–pituitary–testicular (HPT) axis as well [42,43]. The cycle begins with the hypothalamus secreting gonadotropin-releasing hormone (GnRH), which stimulates the anterior pituitary to release the gonadotropins: luteinizing hormone (LH) and follicle-stimulating hormone (FSH). The synthesis of FSH and LH is primarily controlled by the transcription of their distinct beta-subunits, *Fshb* and *Lhb*, which in turn is dependent on the frequency of GnRH pulses, with low frequency favoring FSH and high frequency LH production [44]. Crucially, the pulsatile rather than continuous nature of GnRH signaling is essential for the proper production of both hormones.

In the testes, FSH and LH act through their own specific transmembrane receptors, FSH-R and LH-R. FSH-R is mainly found on Sertoli cells inside the seminiferous tubules, while LH-R is located on Leydig cells in the interstitial tissue. The binding of LH to its receptor activates G protein signaling and increases the levels of cyclic adenosine 3′,5′-monophosphate (cAMP), which stimulates the translocation of cholesterol into mitochondria for conversion into pregnenolone—by the enzyme CYP11A1—and then into testosterone—by the action of 3β-HSD [45]. Synthesized testosterone is released into the bloodstream, reaching systemic circulation and locally, influencing spermatogenesis primarily by regulating factors produced by Sertoli cells (acting on the androgen receptor and exerting these effects along with the previously mentioned hormone FSH) [46]. Testosterone regulates its production through negative feedback mechanisms, with high levels of this hormone suppressing GnRH and gonadotropins release.

In women, the HPGn axis—also designed as hypothalamic–pituitarian–ovarian (HPO) axis—functions similar than in men; Pulsatile GnRH cycles from the hypothalamus influences LH and FSH production in the pituitary gland, reaching the ovarian follicles through systemic circulation. Then, the ovarian production of female sex hormones follows the two-cell, two-gonadotropin model, which describes the cooperative interaction between theca and granulosa cells in steroid hormone synthesis [47]. This model emphasizes the functional specialization of these cell types and their complementary roles in producing the full spectrum of ovarian hormones. Theca cells, located in the outer layer of the follicle, respond to LH stimulation by producing pregnenolone from cholesterol by CYP11A1 as well. Then, depending on various factors such as the phase of the menstrual cycle various pathways can be reported [48]: In the initial phases of the menstrual cycle, pregnenolone undergoes further metabolism to produce A4 and testosterone, which are received by the granulosa cells and converted into estrogens under FSH stimulation. This process is catalyzed by the enzyme aromatase (CYP19A1), which catalyzes the conversion of A4 to estrone and testosterone to estradiol [49]. Efficient hormone production in the ovary depends on the close interaction between theca and granulosa cells, aided by gap junctions and regulated by changing levels of LH and FSH during the menstrual cycle. Alternatively, following ovulation in the luteal phase, the remnants of the ovulated follicle undergo luteinization to form the corpus luteum, a temporary endocrine structure that produces progesterone and estradiol, but not testosterone [50]. Thus, LH specifically stimulates theca cells within ovarian follicles to convert cholesterol into androgens, primarily A4 and testosterone, through a series of enzymatic reactions to be used by the granulosa cells or to reach systemic circulation [51].

In humans, testosterone in the bloodstream is transported bound either to serum albumin (SA)—53 to 55% of the total—or sex hormone-binding globulin (SHBG)—43 to 45%—with the remaining proportion being free [52]. SA acts as a quick-release reservoir that regulates local testosterone concentrations, while SHBG binds more tightly and controls long-term availability of this hormone [53]. Free testosterone, despite only representing a minor portion of total testosterone (approximately 2 to 4%), is believed to be the metabolically active fraction [54]. In general, testosterone levels in healthy men after puberty are ≥300 to 916 ng/dL—with proper references ranges varying with age—whereas in women, the normal testosterone level is around 15–46 ng/dL [55,56].

Testosterone synthesis in the testes increases approximately 30-fold after puberty, and for this reason, adult men typically have 15 to 20 times more circulating testosterone than children or women [57]. In premenopausal women, about 25% of circulating testosterone comes from the ovaries, another 25% from the adrenal glands, and the remaining 50% is produced through the conversion of precursors like A4 in peripheral tissues such as the liver and prominently, the adipose tissue [58]. The levels of serum testosterone in women are also dependent on menstrual cycle. Most studies show a mid-cycle rise in testosterone in women, more evident in younger women, peaking around ovulation due to increased ovarian production regulated by LH, not adrenal sources [59]. Conversely, serum testosterone levels are found to be low in the early follicular phase and the whole of the luteal phase. In postmenopausal women, the ovaries contribute a greater share of testosterone, around 50%. It is worth mentioning that enhanced production of testosterone by hyperresponsive theca cells is a major feature of polycystic ovary syndrome (PCOS), where dysregulation of the HPGn axis leads to excessive LH-driven androgen production [60]. However, in this article we will only focus on normal and reduced production of testosterone reduction in men, although excessive levels of this hormone is also a topic of great relevance in biological and clinical research.

### 3.2. Mechanisms of Action and Metabolism of Testosterone

Testosterone exerts its physiological effects in both men and women through a network of interconnected mechanisms, primarily involving two distinct signaling pathways that regulate gene expression and cellular activity throughout the body. The classical or genomic pathway begins when testosterone diffuses into the cell and binds to androgen receptors (AR) that are initially bound to heat shock proteins (HSPs) in the cytoplasm [61]. This interaction induces a conformational shift in the receptor, leading to its release from chaperone proteins and subsequent translocation into the nucleus. Once there, the AR forms a dimer and binds to androgen response elements (AREs) within the promoter regions of target genes [62]. This process typically initiates transcriptional changes after 30 to 45 min and governs genes associated with sexual differentiation, muscle mass, bone health, and metabolic regulation. In parallel, testosterone can also signal through a non-genomic or rapid pathway that does not require direct modulation of gene transcription. This alternative mechanism involves the activation of membrane-associated receptors and intracellular signaling cascades—such as MAP kinase and CREB pathways—within minutes of hormone exposure. These rapid effects are mediated through G-protein-coupled receptors and involve phosphorylation events facilitated by kinases including MEK/Akt and Src [63,64].

Testosterone can either directly exert effects on target tissues or being transformed into different metabolic products. In androgen-sensitive tissues—such as the prostate, skin, hair follicles, and brain—testosterone is irreversibly reduced by 5α-reductase to form DHT, a more potent androgen that binds the AR with approximately 5 to 10-fold greater affinity than testosterone and mediates local paracrine and intracrine signaling [65]. In adipose tissue, bone, and the brain, aromatase converts a small fraction of circulating testosterone into 17β-estradiol, a key estrogen with many physiological benefits [66].

Although AR is ubiquitously expressed in almost all tissues of men and women and has highly specific functions for each organ, its expression is particularly prominent in androgen-sensitive organs, such as the prostate, epididymis, seminal vesicles, and testes, where it is essential for male sexual differentiation and genital development [67]. In women, there are ARs in various reproductive tissues, including the ovaries, uterus, endometrium, cervix, placenta, vagina, vulva and breast, fulfilling critical roles in these structures [68]. Non-reproductive tissues with high expression of AR include the bones, skeletal muscle, heart, vascular smooth muscle, kidney, pulmonary epithelial cells and the adipose tissue [62,69], along with the central nervous system, prominently in the arcuate nucleus and other medial basal region of the hypothalamus, the bed nucleus of the stria terminalis and amygdala in limbic pathway, the hippocampus, and the temporal lobe—key structures involved in the modulation of mood, behavior and cognitive functions [70].

Finally, testosterone undergoes extensive Phase I metabolism primarily in the liver through cytochrome P450 enzymes, especially CYP3A4, which catalyze hydroxylation reactions such as 6β- and 16β-hydroxylation [71,72]. DHT, though mainly formed in peripheral tissues, is also metabolized in the liver through reduction by aldo-keto reductases like AKR1C2, producing inactive metabolites such as 3α-androstanediol [73]. Both testosterone and DHT metabolites are then conjugated in the liver via glucuronidation by UGT2B15 and UGT2B17, facilitating their elimination through the urine [74].

**Figure 1:**
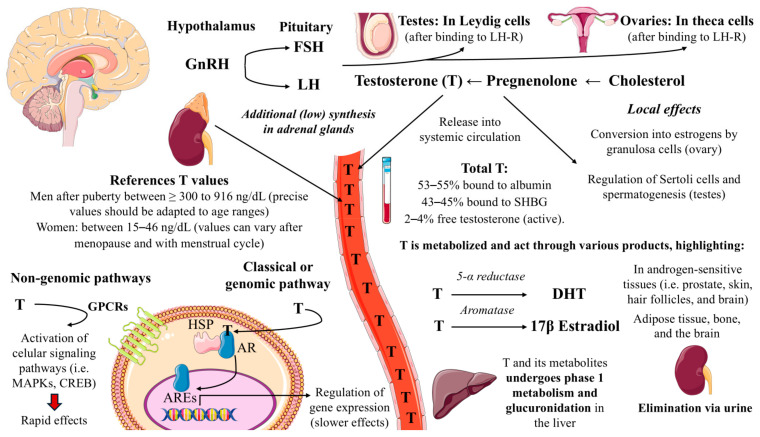
Schematic representation of testosterone (T) biosynthesis, circulation, and mechanisms of action. Cholesterol is converted to pregnenolone and subsequently to testosterone in Leydig cells (testes) and theca cells (ovaries, following luteinizing hormone receptor [LH-R] activation), with additional minor synthesis in the adrenal glands. Testosterone is released into systemic circulation, where ~53–55% is bound to albumin, 43–45% to sex hormone–binding globulin (SHBG), and 2–4% remains as free, biologically active testosterone. Reference serum values are ≥300–916 ng/dL in men after puberty and 15–46 ng/dL in women (with variability depending on menstrual cycle and menopause status). Testosterone exerts its effects via genomic/classical pathways, binding to androgen receptors (AR) stabilized by heat shock proteins (HSP), which then interact with androgen response elements (AREs) to regulate gene transcription. Non-genomic pathways include activation of membrane-bound G protein–coupled receptors (GPCRs), triggering intracellular signaling cascades such as mitogen-activated protein kinases (MAPKs) and cAMP response element–binding protein (CREB). Testosterone is metabolized into dihydrotestosterone (DHT) via 5-α-reductase in androgen-sensitive tissues (e.g., prostate, skin, hair follicles, brain), and into 17-β-estradiol by aromatase in adipose tissue, bone, and brain. Hepatic phase I metabolism and glucuronidation lead to urinary excretion.

In Figure 1 we provide a summary of testosterone synthesis, circulation and mechanism of action explored in this section.

### 3.3. Physiological Functions of Testosterone

As aforementioned, testosterone is a critical hormone with pleiotropic functions in the body. It exerts various regulatory actions and decisively influences metabolic health, the immune system, and cardiovascular, reproductive, neurological, integumentary, and musculoskeletal functions, with equally relevant effects on mental health and psychological functioning. In this section, we will focus on the physiological role of testosterone in both men and women.

#### 3.3.1. Metabolic Regulation

Testosterone serves as a primary anabolic hormone, promoting muscle protein synthesis (MPS) and hypertrophy through direct binding to androgen receptors in muscle cells [4,55]. The anabolic effects are mediated through enhanced neuromuscular function, including improved muscle coordination and force production. Mechanistically, the effects of testosterone on muscle anabolism are achieved together with the growth hormone (GH), exerting complementary and synergic actions. In more detail, testosterone leads to augmented levels of GH by modulating the pituitary growth hormone response to GHRH and favors the GH- insulin-like growth factor 1 (IGF-1) signaling, although testosterone alone also acts by other independent pathways [75,76]. Testosterone demonstrates potent anabolic effects in women through stimulation of muscle protein synthesis as well [77,78,79]. However, research indicates that women maintain higher baseline protein synthesis rates than men across the lifespan, regardless of testosterone levels [77,80], supporting the greater effects and dependence of this hormone in men.

Testosterone exhibits profound effects on glucose metabolism and insulin sensitivity. Clinical studies demonstrate that higher testosterone levels correlate with improved insulin sensitivity, lower HbA1c levels, and reduced stimulated glucose levels during oral glucose tolerance tests [81,82]. Testosterone influences glucose metabolism through non-genomic insulin-like mechanisms, inducing GLUT4 translocation to cytoplasmic membranes and activating AKT, ERK, and mTOR signaling pathways [83].

Regarding lipid metabolism, testosterone decreases total cholesterol, low-density lipoprotein, and triglycerides while increasing high-density lipoprotein [84]. It also inhibits lipoprotein lipase activity in abdominal adipose tissue, reducing triglyceride uptake in central fat depots [85]. As a reflection of its metabolic relevance, it is well-known that testosterone deficiency is strongly associated with metabolic syndrome, with hypogonadism prevalence reaching up to 50% in men with type 2 diabetes mellitus [86].

Testosterone significantly influences body composition by promoting lean body mass and reducing visceral adiposity [87]. The hormone affects fat distribution through stimulation of β-adrenergic-induced lipolysis and inhibition of central fat accumulation [85]. Lower testosterone levels are associated with increased visceral fat content and elevated fatty liver index, while testosterone replacement therapy reduces waist circumference and overall body weight [81].

The relationship between testosterone and metabolic health in women is complex and differs significantly from men. In women not taking contraceptives, testosterone levels correlate with body fat insulin resistance and higher glucose concentrations [88]. Testosterone therapy in obese premenopausal women has also shown minimal effects on plasma lipid concentrations and hepatic VLDL metabolism, with only small decreases in HDL cholesterol observed [78]. This paradoxical relationship may contribute to the metabolic dysfunction observed in conditions like PCOS, where hyperandrogenism is associated with insulin resistance and type 2 diabetes risk [88,89].

#### 3.3.2. Immune Function

Testosterone exhibits complex immunomodulatory effects, generally producing anti-inflammatory actions while selectively downregulating certain immune responses [90,91]. These effects are primarily performed through the regulation of the AR signaling, which involves direct transcriptional regulation of immune-related genes. More specifically, testosterone suppresses NF-κB signaling and reduces pro-inflammatory cytokines including TNF-α, IL-6, and IL-1β [90,92]. Recent evidence demonstrates that testosterone therapy can induce epigenetic adaptations in immune cells, particularly through increased transcription factor activity at canonical NF-κB binding sites in T cells and NK cells [93]. This epigenetic modulation suggests that testosterone’s immunomodulatory effects may have long-lasting consequences on immune cell function beyond immediate transcriptional changes.

Regarding its specific role on the innate immunity, testosterone exhibits dual effects on monocyte and macrophage function, depending on the specific context and concentration. While testosterone generally promotes anti-inflammatory responses, it can also enhance certain pro-inflammatory cytokine production in monocytes. Studies demonstrate that testosterone potentiates monocyte responses, leading to increased production of TNF-α, IL-6, and IL-15 [93]. However, testosterone simultaneously reduces IL-1β production and promotes macrophage polarization toward the anti-inflammatory M2 phenotype through Gαi and Akt signaling pathways [94,95]. In neutrophils, testosterone modulates the generation of reactive oxygen species and fulfills a critical role in neutrophil differentiation and function [96,97]. Testosterone orchestrates dendritic cell (DC) function by regulating antigen presentation and co-stimulatory molecule expression [98]. Likewise, it can also affect the complement system by regulating complement-producing cells and increasing complement inhibitors (CD46, CD55, CD59, clusterin) in reproductive tissues, supporting immune privilege [99]. Generally, higher testosterone suppresses complement activity, though in certain scenarios it enhances complement-mediated bacterial killing, highlighting context-dependent effects [100].

In the adaptive immune system, testosterone selectively suppresses T cell–mediated immunity by inhibiting Th1 (reduced STAT4 and IFN-γ) and Th17 (decreased IL-17 and Th17/Treg ratio) differentiation, while promoting regulatory T cell (Treg) development via direct Foxp3 upregulation and through related orphan receptor gamma (RORγt) downregulation, thus enhancing immune tolerance specially in women [97,101,102,103]. Additionally, testosterone modulates CD8+ T cells, increasing their numbers and cytotoxic potential (↑granzyme B) in certain contexts [93,104]. Testosterone can affect both antibody production and the differentiation of B cell subsets, with implications for humoral immunity [97,105].

The immunomodulatory effects of testosterone exhibit significant sex-specific variations. The responsiveness of female immune cells to testosterone appears to be enhanced in low-androgen environments, while male immune cells from high-testosterone environments show reduced responsiveness [91,101].

#### 3.3.3. Reproductive Functions

Testosterone is essential for maintaining spermatogenesis and male fertility [106,107]. The hormone regulates multiple critical processes within the seminiferous tubules, including maintenance of the blood-testis barrier, completion of meiosis, Sertoli-spermatid adhesion, and sperm release. Testosterone concentrations in the testes are 25 to 125-fold higher than serum levels, indicating the critical importance of local androgen action [107].

At the cellular level, testosterone activates genes in Sertoli cells that promote spermatogonial differentiation and supports the complex process of spermiogenesis [106]. Without adequate testosterone stimulation, spermatogenesis arrests at the meiotic stage, and mature sperm cannot be released from Sertoli cells, resulting in infertility [61]. Beyond spermatogenesis, testosterone maintains overall male reproductive capacity by supporting the development and function of accessory sex organs including the prostate, seminal vesicles, and epididymides [108]. Thus, testosterone ensure proper sperm maturation and transport through the male reproductive tract, making it indispensable for male fertility.

Testosterone plays a crucial role in regulating sexual desire and function through effects on hypothalamic regions involved in sexual motivation [4]. Testosterone significantly influences libido in both men and women, with lower testosterone levels consistently associated with decreased sexual desire and activity [30,109]. In contrast, clinical studies demonstrate that testosterone therapy can restore libido and improve sexual function in men with documented hypogonadism [4], hence supporting the relevance of testosterone in sexual behavior.

#### 3.3.4. Cardiovascular System

Testosterone influences cardiovascular physiology through vasodilation, improved endothelial function, and enhanced cardiac contractility [84,110]. For instance, testosterone can shorten heart-rate corrected QT intervals and demonstrates acute systolic blood pressure-lowering effects [110,111] while enhancing endothelium-dependent and independent brachial artery vasodilation through mechanisms involving potassium channel activation and calcium antagonistic effects [84,111].

Testosterone also stimulates red blood cell production through multiple mechanisms, including erythropoietin (EPO) stimulation and iron metabolism regulation, mainly by suppressing hepcidin and upregulating ferroportin expression [112,113,114]. Clinical studies show that testosterone administration increases EPO levels by approximately 58% and shifts the EPO-hemoglobin relationship curve, establishing a new physiological set point [112]. Testosterone also expands common myeloid progenitors and improves red blood cell survival, collectively increasing hemoglobin, hematocrit, and total red cell mass [114]. These effects are dose- and age-dependent and occur independently of conversion to DHT, thus reflecting a recalibration of the erythropoietic regulatory system [113].

#### 3.3.5. Cognitive and Psychological Effects of Testosterone

Testosterone crosses the blood–brain barrier and exerts neuroprotective effects, including delayed nerve cell death, improved nerve regeneration, and anti-inflammatory actions on neural tissues [115]. For instance, past works have demonstrated that testosterone can prevent neurodegeneration by reducing β-amyloid accumulation, a hallmark of Alzheimer’s disease while improving synaptic signaling, and counteracting neuronal death [116]. In parallel, clinical studies demonstrate that testosterone supplementation can enhance spatial memory, verbal memory, and global cognitive function [115,117,118]. However, these promising results are mainly reported in aging men with both normal and low testosterone states, with and without baseline cognitive dysfunction [119], whereas the cognitive benefits of testosterone for younger men appears to be notably less pronounced [120].

Testosterone profoundly influences brain neurotransmitter systems, particularly dopamine and serotonin pathways that regulate mood, motivation, and reward-seeking behavior [121,122]. Testosterone enhances dopamine signaling in reward-related brain regions, particularly the nucleus accumbens, which increases motivation for status-seeking behaviors and social rewards [123]. This hormonal influence on dopaminergic pathways creates a feedback loop where social success generates both psychological satisfaction and hormonal reinforcement for continued status-oriented behavior. Simultaneously, testosterone affects serotonergic systems that regulate mood, aggression, and social cognition. Critically, the balance between the influences of these neurotransmitters determines whether social behaviors manifest as prosocial cooperation or antisocial dominance [124].

The role of testosterone on behavior is crucially influenced by other hormones such as cortisol. Research demonstrates that testosterone administration can impair cognitive empathy, particularly the ability to infer emotions and mental states from facial cues, but this effect depends on both social context and individual differences in stress responsiveness [125,126]. In high-stress situations with elevated cortisol, testosterone may actually enhance certain aspects of empathy and prosocial behavior, whereas high testosterone and low cortisol report lower levels of empathy [127]. These findings suggest that psychosocial stress modulates how testosterone influences social cognitive processes. Additionally, the role of testosterone is even more complex and is also influenced by its interaction with other neuromodulators such as the nonapeptides oxytocin (OT) and arginine vasopressin (AVP) [128]. Collectively, the integration of testosterone with the different neurotransmitters and neuromodulators further support that testosterone itself is not “causative” of any “good” or “bad” behaviors but correlate with specific and even contrary behaviors depending on the scenario. This context-dependent regulation helps explain why testosterone can promote both aggressive and cooperative behaviors depending on whether the social environment favors dominance assertion or strategic alliance-building [129,130].

Finally, research reveals that testosterone administration results in anxiolytic and antidepressant effects across multiple studies. Meta-analyses demonstrate that testosterone treatment produces moderate but clinically significant reductions in depressive symptoms, with effect sizes translating to meaningful improvements on standardized depression scales, as demonstrated by Walther et al. [131]. According to these authors, the mood-regulating effects of testosterone are dose-dependent, with higher dosages (above 500 mg/week) showing more robust antidepressant effects than lower doses. Importantly, these benefits appear to extend to both hypogonadal and eugonadal men. Similarly, the anxiolytic effect of testosterone also seems to be dose-dependent according to animal models, with various relevant mechanisms explored such as the conversion to DHT and its 3-alpha metabolites, activation of AR, and modulation by endogenous or exogenous opioids [132].

#### 3.3.6. Bone Health

Testosterone exerts significant effects on bone health through direct actions on osteoblasts, osteoclasts, and osteocytes [55]. It also periosteal bone formation during puberty and maintains bone density throughout adulthood. Research demonstrates that testosterone directly inhibits osteoclast formation and bone resorption at physiological concentrations, while also regulating osteoblastogenesis through suppression of IL-6 activity [133,134]. Testosterone deficiency results in decreased bone mineral density and increased fracture risk [135]. This effect is particularly relevant in women, as testosterone exerts significant effects on bone metabolism in women through direct actions on bone cells and conversion to estradiol [136,137]. Research demonstrates a positive association between serum testosterone levels and lumbar bone mineral density in postmenopausal women, with benefits observed up to testosterone levels of 30 ng/Dl [136]. This relationship suggests that increasing testosterone levels in women with low serum concentrations (<30 ng/dL) may improve bone health outcomes. Indeed, clinical studies show that testosterone pellet therapy, alone or combined with estradiol, significantly improves spine and hip bone mineral density in postmenopausal women [138,139].

#### 3.3.7. Effects on Gut Microbiota

Testosterone contributes to gastrointestinal health by supporting normal motility, reducing inflammation, and maintaining gut barrier integrity. Adequate testosterone levels help ensure proper colonic transit, while low levels are linked to slower transit, constipation, and increased risk of disorders such as inflammatory bowel syndrome (IBS) and bowel dysfunction, as demonstrated in preclinical models [140].

But perhaps one of the most interesting effects of testosterone in the digestive system is related to its action on gut microbiota. Emerging research reveals that testosterone significantly impacts the composition and function of the gut microbiome. A systematic review conducted by Pakpahan et al. [141] detected that multiple studies indicate a bidirectional relationship between the gut microbiome and testosterone levels in men. They found that specific taxa, including Ruminococcus, Acinetobacter, Dorea, and Megamonas, are positively correlated with testosterone, while Bacteroides show negative associations. Likewise, they reported that gut microbiome diversity is generally higher in men with elevated testosterone, and microbial metabolism—including deglucuronidation and steroid-processing enzymes—can directly influence androgen bioavailability. The proposed mechanisms included interactions that affect systemic metabolism, SHBG levels, and potentially the HPGn axis.

In preclinical models, Moadi et al. [142] found that before puberty, the microbiome shows limited exposure to sex hormones, making it responsive to in vitro testosterone or DHT, which increase Bacteroidetes and Firmicutes while reducing Proteobacteria. After puberty, they observed that male and female microbiomes diverge, with males showing higher Clostridiales and females higher Turicibacter; testosterone supplementation in females shifts their microbiome toward a more “male-like” composition, decreasing Bifidobacterium and altering metabolites such as L-citrulline and cysteine-s-sulfate. Recently, Harris et al. [143] demonstrated that testosterone therapy in transgender individuals did not affect gut microbiome composition in short term, it leaded to a shift in the abundance of genes related to microbial metabolism, particularly those affecting glutamate and glutamine utilization. They hypothesized that these changes likely increase host competition for dietary glutamate, contributing to elevated plasma arginine and citrulline levels, linking testosterone exposure to modifications in host-microbiome metabolic interactions.

These findings support the concept of a “testobolome,” analogous to the estrobolome [144], representing in this case the subset of gut microbial genes, enzymes, and pathways that modulate testosterone and other androgens. Recognizing the testobolome highlights the bidirectional interaction between testosterone and the gut microbiome and provides a framework for future studies on microbiome-mediated regulation of androgen-dependent physiology and disease.

#### 3.3.8. Renal Function

The relationship between testosterone and renal function presents a complex picture with both beneficial and potentially concerning effects. Studies from testosterone administration show that long-term therapy in hypogonadal men is associated with improved glomerular filtration rate (GFR) and overall renal function [145] whereas acute administration may transiently reduce estimated GFR due to increased creatinine from muscle gains rather than true renal impairment [146]. Testosterone modulates the renin–angiotensin–aldosterone system (RAAS), maintaining higher AT_1_R/AT_2_R ratios and enhancing vascular responsiveness to angiotensin II, which can influence blood pressure and cardiac remodeling [147]. Despite these complex effects, testosterone may exert nephroprotective benefits over time, improving outcomes in chronic kidney disease via better muscle mass, anemia management, and metabolic health [148,149].

#### 3.3.9. Respiratory System

Testosterone contributes to respiratory physiology by enhancing lung function and respiratory muscle strength, leading to improvements in forced expiratory volume in 1 s (FEV_1_) and forced vital capacity (FVC) [150]. It also induces rapid bronchodilation through non-genomic mechanisms, including nitric oxide-mediated airway relaxation and upregulation of potassium channels (Kᵥ1.2/1.5) and P2Y_4_ receptors [151,152]. These mechanisms enhance airway smooth muscle relaxation and may protect against bronchospasm, airway hyperreactivity, and inflammatory lung diseases [153]. Additionally, testosterone deficiency is linked to impaired pulmonary immune regulation and increased susceptibility to emphysema, suggesting that higher testosterone levels contribute to maintaining respiratory and pulmonary health [150,154].

#### 3.3.10. Effects on Skin and Hair

Testosterone exerts profound effects on skin and hair physiology in both men and women through complex androgenic pathways involving DHT conversion and sebaceous gland stimulation. In men, testosterone causes skin to be approximately 10–20% thicker than women, while simultaneously stimulating sebaceous glands to produce up to four times more sebum, resulting in oilier skin texture and larger pores [155,156,157]. The well-established effects of testosterone on skin thickness are supported by novel mechanisms involving enhanced collagen synthesis and improved dermal architecture. Testosterone directly stimulates collagen production in dermal fibroblasts through multiple pathways, including upregulation of growth arrest-specific protein 6 (Gas6) and Axl signaling, promoting anti-aging effects by modulating tissue inhibitor of metalloproteinase-2 (TIMP-2) expression and reducing of membrane type-1 metalloproteinase (MT1-MMP), creating an optimal balance that prevents excessive collagen degradation [158]. In women, testosterone produces similar but less pronounced effects: elevated testosterone levels increase sebum production and contribute to acne development, with 72% of women with acne showing excess androgen hormones [159], while hyperandrogenic conditions like PCOS are associated with increased facial hair growth and male-pattern hair loss through the same DHT-mediated mechanisms [160]. 

**Figure 2:**
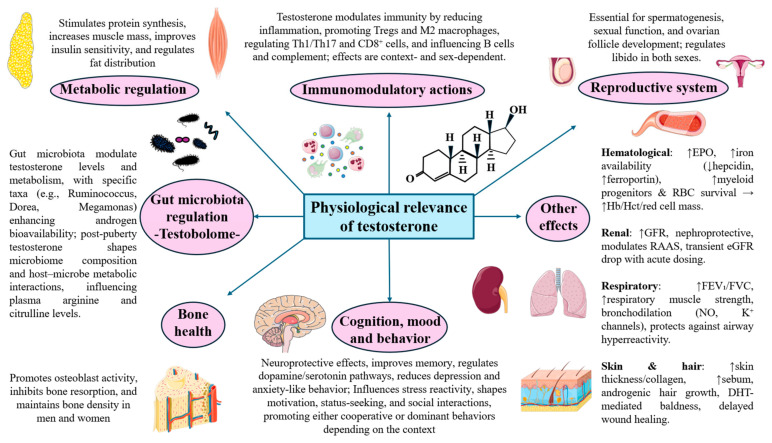
Physiological relevance of testosterone across multiple systems. Testosterone regulates metabolism by stimulating protein synthesis, increasing muscle mass, improving insulin sensitivity, and modulating fat distribution. It exerts immunomodulatory effects by attenuating inflammation, promoting regulatory T cells (Tregs) and M2 macrophages, modulating Th1/Th17 and CD8^+^ T cells, and influencing B cell and complement activity in a sex- and context-dependent manner. Within the reproductive system, testosterone is essential for spermatogenesis, sexual function, ovarian follicle development, and libido regulation in both sexes. Neurocognitive and behavioral effects include neuroprotection, enhanced memory, regulation of dopamine and serotonin pathways, reduced depression and anxiety-like behaviors, modulation of stress reactivity, and shaping of motivation and social interactions. In bone health, testosterone promotes osteoblast activity, inhibits bone resorption, and preserves bone density. Additional systemic roles include hematopoiesis (↑ erythropoietin [EPO], ↑ iron availability via ↓ hepcidin and ↑ ferroportin, ↑ myeloid progenitors, and red blood cell [RBC] survival), renal protection and regulation of the renin–angiotensin–aldosterone system (RAAS), respiratory benefits (↑ forced expiratory volume in one second/forced vital capacity [FEV_1_/FVC], enhanced respiratory muscle strength, bronchodilation), and skin/hair modulation (↑ dermal thickness and collagen, ↑ sebum, androgenic hair growth, dihydrotestosterone [DHT]-related baldness, delayed wound healing). Gut microbiota also regulates testosterone metabolism (“testobolome”) and are in turn shaped by testosterone post-puberty, influencing host–microbe metabolic interactions.

While testosterone’s effects on skin structure are generally beneficial, its influence on wound healing presents a paradoxical picture. Testosterone significantly inhibits cutaneous wound healing through androgen receptor-mediated pathways, creating enhanced inflammatory responses and delayed tissue repair [161]. Likewise, regarding hair, DHT binding to androgen receptors in genetically susceptible hair follicles, causing miniaturization and eventual male pattern baldness—notably, it is the follicle sensitivity to DHT rather than absolute testosterone levels that determines hair loss [162]. Both sexes experience similar DHT-mediated hair follicle miniaturization when genetically predisposed, though women typically present with different patterns of hair thinning compared to the characteristic male pattern of receding hairlines and crown baldness [162]. Collectively, the different physiological functions of testosterone are summarized in Figure 2.

## 4. Evidence, Causes and Consequences of Testosterone Decline in Modern Times

### 4.1. Evidence of Testosterone Decline

The evidence for a global and age-independent (secular) decline in testosterone levels among men has been accumulating for decades, with multiple large-scale studies documenting this concerning trend. One of the most striking aspects of the documented testosterone decline is its age-independent nature, meaning that men of all ages are experiencing lower testosterone levels compared to men of the same age in previous decades. This phenomenon has been observed across the entire adult age spectrum, from adolescents to elderly men [13,163]. A systematic analysis of 1257 studies accounting for 1,064,688 subjects found a significant negative linear regression between testosterone levels and year of measurement, highlighting an annual decline of 0.56% that persisted after adjusting for age and assay methodology [164].

A significant number of studies evaluating this phenomenon has been conducted in the United States. A landmark study published in 2007 from the Massachusetts Male Aging Study involving 1532 men across three data collection waves from 1987 to 2004, demonstrated a substantial age-independent decline in testosterone levels that could not be attributed to observed changes in health and lifestyle characteristics, including smoking and obesity [12]. This longitudinal analysis revealed that population-level declines were greater in magnitude than the cross-sectional declines typically associated with age, indicating that recent cohorts of men have lower testosterone levels at any given age compared to their predecessors.

Another longitudinal U.S. cohort study (Air Force veterans) by Mazur et al. found a dramatic decline: mean total T fell from 638 ng/dL in 1982 to 431 ng/dL in 2002 (a 207 ng/dL drop, over 33%) [18]. This decline far exceeded the roughly 100 ng/dL expected from aging alone, implicating a true generational effect. Mixed-effects analysis confirmed an age-independent decline in testosterone across the 20-year study [18].

A 2021 study analyzing adolescent and young adult men (ages 15–39) using data from the National Health and Nutrition Examination Surveys (NHANES) from 1999 to 2016 revealed a nearly 25% decrease in average total testosterone levels over this 17-year period. Mean total testosterone decreased dramatically from 605.39 ng/dL in 1999–2000 to 451.22 ng/dL in 2015–2016, representing one of the most concerning trends in male reproductive health even after adjusting for body mass index (BMI) [165]. In more detail, they observed that mean total testosterone decreased dramatically from 605.39 ng/dL in 1999–2000 to 451.22 ng/dL in 2015–2016, representing one of the most concerning trends in male reproductive health.

Subsequent research has confirmed this trend across multiple populations. Andersson et al. examined Danish population surveys from 1982 to 1983 through 1999–2001 and similarly reported that age-matched cohorts born later had lower testosterone levels than earlier cohorts [166]. In that study, adjustment for rising BMI largely accounted for the drop in total testosterone, suggesting obesity partly explained the trend, although SHBG continued to rise independently. Perheentupa et al. [167] analyzed Finnish men of various ages in national surveys from 1972, 1977 and 2002. A notable secular trend was identified in levels of testosterone (both total and free), SHBG, and gonadotrophins, with more recently born men showing lower levels compared to age-matched individuals from earlier birth cohorts. For example, in men aged 60–69, serum testosterone dropped from 21.9 nmol/L in those born between 1913 and 1922 to 13.8 nmol/L in those born between 1942 and 1951. These declines remained statistically significant even after adjusting for BMI. The data indicated a birth cohort effect on reproductive hormones in Finnish men that was independent of age.

More recent large database studies also support a secular decline. Chodick et al. (2020) analyzed over 100,000 Israeli men tested between 2006 and 2019 and reported a highly significant age-independent drop in total T across all adult age groups [13]. A Swedish study by Trimpou et al. compared population samples of men in 1995 versus 2008 and found that 2008 men had significantly lower free T than same-aged men in 1995 [168]. Even routine clinical data hint at the trend: Walsh et al. [169] examined trends in testosterone testing, prevalence of low testosterone, and treatment initiation among U.S. Veterans aged 40–89 years in the Northwest (VISN 20) from 2002 to 2011. They observed that the proportion with low T rose from ~35% to 47%, indirectly consistent with a population shift. However, the percentage of men receiving testosterone therapy after a low result slightly decreased, suggesting growing clinical caution despite the rising prevalence of biochemical hypogonadism.

The temporal pattern of decline appears to have accelerated in recent decades. While earlier studies documented gradual decreases from the 1970s through the early 2000s, more recent data suggests the decline has continued unabated into the first and second decades of the twenty-first century. Longitudinal analyses have demonstrated that the decline in mean testosterone over 20 years was at least twice what would be expected from cross-sectional estimates of aging decline alone, indicating that cohort effects significantly compound the natural aging process [18].

Not all studies find a clear secular decline. Nyante et al. [170] used NHANES (1988–1991 vs. 1999–2004) and reported *no significant change* in overall total T for U.S. men over that period. They did, however, note subgroup changes (e.g., SHBG and 3α-diol-G were reduced in young white men, estradiol declined in white and Mexican-American men, and free testosterone increased in young black men). The reasons for discrepant results remain unclear, but differences in sampling and analysis (e.g., controlling for BMI) likely play a role.

In contrast to men, there is no consistent evidence of a generational and secular decline in women’s androgen levels. Changes in hormone levels among women tend to align closely with natural aging rather than reflecting shifting baselines across birth cohorts [171,172]. Significant alterations in androgens often occur due to events like menopause or surgical procedures such as oophorectomy [173], but these are linked to biological aging or medical interventions rather than broader environmental or lifestyle influences. Some smaller studies have reported specific, context-related reductions—such as lower testosterone levels in mothers or in married women—but these variations are more associated with reproductive or social circumstances than with widespread secular trends [174,175]. Overall, unlike the well-documented generational testosterone decline seen in men, current evidence does not indicate a similar pattern in women, whose hormone levels generally follow a gradual, age-related decrease. Because of this and from this point we will exclusively refer to the causes, consequences and potential interventions directed to testosterone decrease in men. However future studies are warranted to define if these secular changes might also be occurring in women as well, particularly given the potentially shared factors contributing to this fact, as it will be subsequently discussed.

In Table 1, a summary of the main studies evidencing the secular decrease of testosterone is provided.

### 4.2. Possible Causes and Potential Consequences of the Secular Testosterone Decline in Men

#### 4.2.1. Causes

The documented global decline in testosterone levels represents a complex multifactorial phenomenon involving interconnected environmental, lifestyle, and societal changes that have emerged over the past several decades. Scientific evidence points to several primary contributing factors that collectively explain this concerning trend, including:

##### Overweight, Obesity and Other Relevant Health Issues

Obesity and being overweight represents one of the most significant and well-established causes of testosterone decline. According to the World Health Organization (WHO), an estimated 2.5 billion adults aged 18 and over were classified as overweight in 2022, including more than 890 million living with obesity [176]. This means that 43% of adults worldwide—43% of men and 44% of women—had excess weight, a notable rise from 1990 when the prevalence was 25%. Despite being generally more prevalent among women and older age groups than among men and younger age groups, compelling evidence supports that male individuals and youths have suffered a more drastic rise in obesity prevalence in recent decades [177]. Indeed, in 2022, approximately 21% of boys aged 5–19 were overweight, compared to 19% of girls [178], supporting the present and growing relevance of obesity as a critical concern of testosterone decline in males. The relationship between excess body fat and testosterone suppression operates through multiple interconnected and bidirectional mechanisms that create a vicious cycle of hormonal dysfunction that favors and are facilitated by excess adipose tissue [179]. Because of this, obese men, especially with BMI > 35–40, can have up to 50% less testosterone than lean men, with other variables such as the weight-adjusted-waist index (WWI) also predicting broader reductions in testosterone levels [179,180,181]. Despite the relevance of this factor, studies have shown that even men who maintained constant weight or lost weight during longitudinal follow-up experienced significant testosterone declines, with mean testosterone declining 117 ng/dL (19%) over 20 years even among men who held their weight constant. This finding challenges the hypothesis that rising obesity rates alone explain the observed decline [18].

Beyond overweight and obesity, a wide range of additional pathologies can partly explain the secular decline in testosterone observed in the general population. Recent evidence suggests that nowadays 6 in 10 young, 8 in 10 midlife, and 9 in 10 older US adults report 1 or more chronic conditions [182], supporting the important role that chronic diseases may have and their potential connection with testosterone. For instance, type 2 diabetes mellitus (T2DM) is also commonly related to reduced testosterone levels, partly due to alterations in SHBG levels and bidirectional associations with visceral fat, muscle, and possibly bone, as low testosterone promotes insulin resistance and metabolic dysfunction, while diabetes and metabolic alterations further suppress testosterone production [183,184]. Patients with various metabolic disorders such as T2DM and obesity (named diabesity) and/or metabolic syndrome tend to present even reduced testosterone levels due to the synergic effects of these disorders [185].

Cardiovascular disease shows similar complex interactions with testosterone levels. Low testosterone is associated with increased cardiovascular mortality, with men in the lowest quartile of testosterone having a 40% increased likelihood of 20-year mortality [22,110,186]. Testosterone deficiency adversely affects multiple cardiovascular risk factors, including promoting central adiposity, dyslipidemia, endothelial dysfunction, and insulin resistance [110,186,187]. In parallel, cardiovascular disease and its risk factors can suppress testosterone production through chronic inflammation and metabolic stress.

Other significant health conditions affecting testosterone include hypogonadism caused by various etiologies: primary hypogonadism from testicular disorders (Klinefelter syndrome, testicular infections, trauma, chemotherapy), secondary hypogonadism from hypothalamic–pituitary dysfunction (tumors, infections, medications like opioids and corticosteroids), and mixed forms combining various mechanisms. Chronic systemic illnesses including end-stage renal disease, HIV infection, inflammatory maladies, and sleep disorders all contribute to testosterone suppression through multiple pathophysiological pathways involving stress response activation, inflammatory cytokine release, and disruption of normal hormonal regulation [188].

##### Exposure to Endocrine-Disrupting Chemicals

Exposure to EDCs—including phthalates, bisphenol A (BPA), per- and polyfluoroalkyl substances (PFAS), and micro/nanoplastics—seem to be a major environmental contributor to declining testosterone levels. Research has demonstrated negative correlations between exposure to these chemicals and testosterone levels, with mixture effect analyses confirming that combinations of environmental chemicals have cumulative impacts on male reproductive hormones [17,189]. Phthalates, commonly found in plastics, inhibit testosterone synthesis in Leydig cells by several mechanisms such as the disruption of key steroidogenic pathways and transcription factors like StAR and CYP17 [189,190]. BPA, present in food packaging and plastics, mimics estrogen, blocks androgen receptors, decreased expressions of steroidogenic enzymes and cholesterol carrier protein in Leydig cells and interferes with LH signaling, reducing testosterone production [191]. In addition, in vivo models also support that high exposure to BPA alters mitochondrial protein expression in mouse testes, disrupting hormone balance and impairing male fertility by activating estrogen receptors, PKA, and MAPK pathways, which in turn affect mitochondrial respiration, ATP production, and promotes apoptosis in testicular cells [192]. PFAS, or “forever chemicals,” accumulate in the body and are linked to impaired sperm function, testicular damage, and hormonal imbalance, likely through oxidative stress and disruption of steroidogenesis [193]. Microplastics and nanoplastics, increasingly detected in human tissues including semen, act as carriers for EDCs and directly damage reproductive cells, disrupt the blood-testis barrier, and trigger oxidative stress [194,195].

Pesticides represent another significant class of EDCs with documented effects on testosterone levels and male reproductive health. Organophosphate pesticides, widely used in agriculture, have been shown to reduce sperm count, concentration, and motility, though effects on testosterone levels remain inconsistent across studies, with some meta-analyses reporting no significant impact on serum testosterone, FSH, or LH levels, suggesting testosterone-independent mechanisms of reproductive toxicity [196]. However, other pesticides demonstrate more direct hormonal effects, with atrazine—the second most widely used herbicide in the United States—showing particularly concerning endocrine disrupting properties as demonstrated by a recent meta-analysis revealing that atrazine exposure significantly decreases serum FSH, LH, and testosterone levels while increasing estradiol and progesterone [197]. Among the mechanistic basis explored in preclinical models, atrazine at environmentally relevant concentrations (as low as 0.1–25 ppb) can directly inhibit Leydig cell testosterone synthesis, enhance aromatase activity and disrupt normal steroidogenesis while affecting hormone signaling pathways [198,199,200]. Organochlorine pesticides including heptachlor and DDT have also been associated with lower testosterone levels in exposed men [201], while other pesticides such as glyphosate can decrease testosterone by up to 35% at relatively low concentrations (1 part per million) [202]. The mechanisms underlying pesticide-induced testosterone disruption include direct inhibition of steroidogenic enzymes, interference with GnRH signaling, oxidative stress induction, and disruption of cellular energy metabolism in testicular tissues [203,204,205].

##### Sedentarism

Lifestyle factors associated with modern society also play crucial roles. Sedentary behavior is one of the critical factors associated with testosterone decline in modern times, as supported by a growing number of studies [15,206,207]. According to recent reports [208], up to one third of the global population do not reach the minimum levels of physical activity recommended by the WHO—at least 150 min of moderate-intensity activity per week, 75 min of vigorous-intensity activity, or an equivalent combination—although the percentage of inactive people may vary depending on geographical differences, age, or if considering sedentarism in the work or at leisure time [209]. The increasing prevalence of sedentary lifestyles contributes to testosterone decline through multiple pathways, including increased body weight, altered metabolic and endocrine function, elevated oxidative stress levels, and disrupted sleep patterns [210]. Additionally, chronic inflammation associated with sedentary behavior shows positive correlations with testosterone deficiency risk, suggesting that the pro-inflammatory environment of modern life may suppress the HPGn axis [34,211,212].

##### Unhealthy Dietary Patterns

Modern unhealthy dietary patterns (such as Western diets) correlate strongly with reduced testosterone levels. Regarding specific macro- and micronutrients it is worth of mentioning that these diets are characterized by high consumption of ultraprocessed foods and drinks (UPFDs), rich in sugar and refined carbohydrates, fried foods, and artificial trans fats, while poor in fruits and vegetables (rich in micronutrients) and high-quality fats and proteins [213]. These types of unhealthy diets are strongly associated with gut dysbiosis and systemic inflammation, along with a lack of essential nutrients that collectively impact on male reproductive health, evidenced by reductions in sperm counts and testosterone levels and raises in reproductive pathologies [214]. For instance, a study of the “fried-processed dietary pattern” found independent negative associations with testosterone levels, with effects potentially mediated through inflammation, insulin resistance, and oxidative stress [215]. Similarly, the offspring of pregnant rats fed with cafeteria diet, a selection of foods emulating highly energetic and palatable human foods, show a significant reduction in testosterone, LH and FSH levels, supporting the deleterious impact of modern unhealthy diets on endocrine health [216]. Regarding specific nutrients, high sugar consumption represents one of the most immediate dietary threats to testosterone levels. Clinical studies demonstrate that glucose ingestion causes acute decreases in testosterone levels lasting even more than two hours after consumption [217,218,219]. The mechanism involves insulin-induced suppression of gonadotropin secretion and direct inhibition of testicular steroidogenesis [220]. Trans fatty acids, on the other hand, directly correlate with lower testosterone levels in clinical studies [221]. These artificial fats disrupt normal cellular membrane function and interfere with steroidogenic enzyme activity, driving to decreased fertility, sperm count, motility and normal morphology and, in extreme cases, arrest of spermatogenesis and testicular degeneration [222]. Thus, unhealthy dietary patterns, characterized by a high intake of these compounds, have a significant impact on testosterone levels, partially explaining the secular decrease in testosterone levels observed in the studies.

##### Sleep Deprivation and Disorders

Sleep deprivation has dramatic effects on testosterone levels: restricting sleep to 5 h per night for just one week reduces testosterone levels by 10–15% [223,224]. Sleep disorders including sleep apnea, circadian rhythm disruption, and poor sleep quality all contribute to testosterone suppression, creating a bidirectional relationship where low testosterone can also impair sleep quality [225]. Modern lifestyle factors contributing to sleep disruption include excessive screen time with blue light exposure, irregular sleep schedules, caffeine consumption, and chronic stress [226]. This reduction occurs through multiple mechanisms including disruption of circadian rhythm regulation, impaired REM sleep, reduced GH release, and increased cortisol production [227,228]. According to previous studies, sleep loss and lower sleep duration are linked to reduced morning, afternoon and 24 h testosterone, whereas they are associated with higher late afternoon and early evening, but not morning or 24 h cortisol [229]. Consequently, the reciprocal changes in testosterone and cortisol with sleep loss disturb catabolic-anabolic signaling and is a relevant mechanism by which sleep loss induces insulin resistance and other concerns.

##### Chronic Stress and Sociocultural Factors

The regulation of testosterone through psychosocial factors represents a complex bidirectional relationship where environmental stressors, social contexts, and psychological states dynamically influence hormone production while testosterone simultaneously shapes behavioral responses to these same factors. This intricate interplay occurs through multiple neurobiological pathways that integrate stress responses, social cognition and motivational systems. The relationship between psychosocial and biological factors could be understood thanks to two critical areas of knowledge: The biopsychosocial model and the psychoneuroimmunoendocrinology (PNIE). The first one was firstly described by George Engel in 1977 in order to integrate psychological and social factors along with biological determinants in order to create a comprehensive framework for health and disease [230]. PNIE—which can also be designed as psychoneuroimmunology (PNI)—is a complementary field of research developed by Robert Ader and Nicholas Cohen in the 1970s, who firstly reported an association between the brain and the immune system, showing that psychological and social factors can influence how the brain and immune system function [231]. With subsequent research and following a growing body of evidence, PNIE now emphasizes that psychosocial factors influence and are influenced by the psyche (P) or mind, which in turn regulates—and is regulated—by the nervous system (N), the immune system (I) and the endocrine system (E). Thus, neither body and mind nor biopsychosocial factors can be dissociated and understood separately, as they are interdependent and dynamic variables in constant communication [232]. In this context, the relationship between testosterone and psychosocial factors can be studied from multiple perspectives.

Psychosocial stress exerts profound effects on testosterone regulation through the HPGn axis, which functions in close coordination with the hypothalamic–pituitary–adrenal (HPA) axis responsible for cortisol production [233]. When individuals encounter stressful situations, the body prioritizes cortisol production over testosterone synthesis since both hormones share the same precursor molecule, pregnenolone. This competitive relationship means that chronic elevation of cortisol from sustained psychosocial stress can significantly suppress testosterone levels by inhibiting GnRH secretion from the hypothalamus and reducing pituitary responsiveness to GnRH pulses [233,234]. Studies suggest that various forms of chronic stress can lead to measurably reduced testosterone concentrations, as demonstrated in animal models [8]. In turn, changes in testosterone influence the acute and chronic response to stress [235], creating a vicious cycle in which testosterone and cortisol changes are mutually affected.

Moreover, various sociocultural factors are implicated in testosterone regulation. Economic inequality and social status dynamics represent sociocultural influences on testosterone regulation, as heightened economic disparities foster competitive environments where men with more advantaged socioeconomic positions demonstrate higher risk-taking behavior and testosterone levels, while people with lower socioeconomic status, chronic financial stress and perceived social disadvantage show reduced testosterone levels, likely through elevated cortisol pathways and associated psychological pressures [236,237]. Social relationship dynamics also play a crucial mechanistic role, with committed romantic partnerships and marriage associated with approximately 21% lower testosterone levels compared to single men [238], whereas no significant differences are observed between single men and those with a new relationship (less than 12 months) [239], reflecting evolutionary adaptations to pair-bonding that down-regulate mating efforts and competitive behaviors. Occupational changes from physically demanding manual labor to sedentary employment could have fundamentally altered testosterone regulation, as suggested by a recent research [240] reporting that men in physically demanding jobs demonstrating 46% higher sperm concentrations and higher testosterone levels compared to desk-based workers, suggesting that the decline in manual occupations contributes to population-level testosterone decreases. The proliferation of digital technology and social media usage can impact on the endocrine system and introduce novel stressors including excessive screen time, sleep disruption from blue light exposure, and impaired social interaction patterns that collectively influence hormonal balance through disrupted circadian rhythms and increased sedentary behavior [241]. Although findings remain heterogeneous, compelling evidence suggests that prolonged exposure to radiofrequency electromagnetic radiation (RF-EMR) from mobile phones and Wi-Fi devices may disrupt male reproductive hormones, particularly testosterone in a dose-dependent manner, supporting recommendations to limit its excessive use [242].

Perhaps one of the most controversial causes to be explored nowadays could be the changing context of social and gender norms. The “reverse relationship” framework suggests that gendered experiences—like norms around masculinity and femininity—can shape testosterone levels according to some previous works [243]. This gender-testosterone pathway involves sociocultural, neurobiological, and evolutionary inputs, with competition being a key context. In modern society, shifting ideas of masculinity and changing social hierarchies may create psychological uncertainty about male identity and status, potentially influencing testosterone in new, culturally driven ways [243]. Another study also found that low testosterone at baseline predicted heightened cortisol reactivity after gender threatening [244], although other authors suggest that high testosterone levels could be related to masculine overcompensation—a theory proposing that men respond to threats to their masculinity with exaggerated displays of stereotypical masculine traits [245]. However, to date, the evidence holds that the relationship between testosterone and masculinity, as well as with the rest of psychosocial dimensions, is complex and context dependent.

##### Medications

Common medications represent a significant yet underappreciated contributor to the secular decline in testosterone levels, with several drug classes demonstrating direct suppressive effects on the HPGn axis through various mechanisms. Preclinical models show that selective serotonin reuptake inhibitors (SSRIs) interfere with testosterone production by disrupting HPA axis function and altering GnRH secretion, with research showing these antidepressants usually reduce testosterone levels [246,247,248,249]. On the other hand, evidence in humans is scarce. Paroxetine and fluoxetine have been tested clinically, with studies showing no consistent changes in serum testosterone, while citalopram, escitalopram, and sertraline remain unexplored in patients [250]. Among other antidepressants, venlafaxine has only isolated case reports linking it to testosterone suppression, duloxetine and desipramine have no clinical data, and studies with amitriptyline, imipramine, and bupropion report either neutral or inconsistent findings [250]. Although men are diagnosed with major depressive disorder (MDD) at half the rate of women and are often undertreated, there is growing evidence suggesting that men may be expressing these concerns in different ways not totally covered in clinical guidelines, which could explain the 3 to 4 times higher risk of suicide or substance abuse patterns [251,252]. Thus, exploring the impact of antidepressants with testosterone levels in men and their association with clinical response represent a critical area of future research to explore.

Opioid medications produce particularly pronounced effects, with studies demonstrating testosterone suppression of nearly 50% in chronic users through inhibition of GnRH release and subsequent reduction in LH and FSH secretion [253,254]. The literature recognizes that although men were significantly less likely than women to report opioid use, they were significantly more likely to report opioid misuse and to misuse prescription opioids. Indeed, approximately 29% of men met misuse criteria and up to 70% of all opioid overdose deaths in 2017 occurred in males, with prescription opioids being responsible for roughly 35% of the total [255,256]. Thus, deepening on the association between opioid prescription, testosterone and male physical and mental health is a central topic of study for future research.

Other medications with proven effects on testosterone downregulation are statins. A significant number of systematic reviews and clinical studies show that statins appear to reduce in men total testosterone by 0.66 nmol/L, as well as free and bioavailable testosterone, although probably not enough to cause a drop below the normal range in healthy individuals [257,258,259]. However, as the prescription of statins have been increasing in past years [260], considering its potential effects on reducing testosterone levels also represent an important area of research in order to consider what kind of patients.

Chronic glucocorticoid therapy significantly suppresses the HPG axis through direct inhibition of GnRH neurons and increased cortisol-mediated feedback inhibition, with studies showing important reductions in plasma testosterone levels during hydrocortisone treatment [261,262,263]. Long-term oral glucocorticoid prescriptions increased from 3.3% of adults in 2006 to 4.3% in 2017, indicating growing cumulative exposure [264]. Antihypertensive medications, particularly beta-blockers and thiazide diuretics, have been associated with testosterone reduction through effects on the HPGn axis and alterations in bioavailable testosterone via increased SHBG binding [265,266,267]. The prevalence of antihypertensive drug prescriptions in men rose from 7.8% in 1988 to 21.9% in 2018, highlighting a substantial increase in treatment of hypertension [268].

The clinical significance of medication-induced reductions in testosterone levels extends beyond isolated hormonal changes, as these drugs are commonly prescribed for conditions that themselves are associated with low testosterone—including depression, chronic pain, cardiovascular disease, and metabolic disorders—creating a potentially synergistic effect that may accelerate the age-related decline in testosterone levels observed in contemporary male populations.

##### Substance Abuse

Substance abuse can also be an important contributing factor to reducing testosterone levels globally. Indeed, the literature recognize that substance abuse and misuse of prescription drugs is generally more prevalent in males than in females. For instance, men are 2 to 3 times more likely than women to meet criteria for a substance use disorder (SUD) in their lifetime [269]. In a review conducted by Duca et al. [270] they reported that heavy alcohol consumption (but not moderate) impairs testosterone production and spermatogenesis through multiple mechanisms, including hormonal disruption and oxidative stress. In a similar way, amphetamines, opioids and anabolic-androgenic steroids (AAS) were also negative modulators of testosterone by suppressing the HPT axis and affecting testicular function, whereas the effect of other drugs such as cocaine or cannabis remain inconclusive. The effect of tobacco in testosterone appears to be more complex. Indeed, it seems that smokers tend to have higher testosterone levels than non-smokers [271]. More detailly, Wang et al. [272] observed that smoking has a positive and an independent effect on testosterone levels but depending on the context. It seems that tobacco can acutely stimulate GnRH and LH production and reduce the conversion of testosterone into estradiol, aiding to explain the positive correlation observed in smokers. Conversely, they reported an inverse association between years of smoking and testosterone levels, possibly due to the detrimental effects of long-term cigarette smoking on testosterone synthesis by impairing Leydig cells. Similarly, caffeine is another substance associated with increased levels of testosterone in athletes in a dose and form-dependent manner [273,274]; however other studies show little or no effect of caffeine on testosterone levels in trained and healthy adult men [275,276] and even negative associations between caffeine metabolites and testosterone levels [277], suggesting the need of further studies to understand the effect of caffeine on testosterone levels.

##### Infections and Dysbiosis

Viral infections represent a critical yet frequently overlooked determinant of testosterone suppression and male infertility, particularly evidenced by the SARS-CoV-2, the virus responsible for the coronavirus disease 19 (COVID-19). A recent systematic review and meta-analysis published by Ashonibare et al. [278] has found that SARS-CoV-2 significantly reduced ejaculate volume, sperm count, concentration, viability, normal morphology, and total and progressive motility, with these changes linked to a significant reduction in circulating testosterone level, but a rise in estrogen, prolactin, and LH levels. Mechanistically, the reduction in testosterone levels by SARS-CoV-2 seems to be through direct infection of Leydig and Sertoli cells, with disease severity correlating with testosterone reduction in symptomatic patients [279]. In turn, greater reductions in testosterone has been associated with increased mortality risk in other studies, regardless of age or presence of comorbidities [280]. In survivors, although testosterone levels tend to rise ≥3 months after recovery in the majority of cases, Salonia et al. [281] found that almost 10% of patients showed reduced testosterone levels when compared to the time of hospitalization after a follow-up of 7 months, and that approximately 55% of their cohort presented total testosterone levels suggestive of hypogonadism after this period. In this sense, Sauve et al. [282] reported that persisting hypotestosteronemia could be observed in some patients even more than 1 year after presentation with COVID-19 due to the SARS-CoV-2 neuroinvasion of GnRH neurons and tanycytes. Collectively, these studies indicate that SARS-CoV-2 appears to modulate testosterone levels through several mechanisms both at the time of infection and even more than one year after its clinical presentation.

Similarly, other respiratory viruses, including H7N9 avian influenza and seasonal influenza strains, similarly suppress circulating testosterone with significantly worse clinical outcomes [283], whereas mumps virus directly inhibits Leydig cell testosterone secretion by targeting the CYP17A1 steroidogenic enzyme [284]. The human immunodeficiency virus (HIV) is also linked to reduced testosterone levels in people living with this chronic infection, probably due to direct toxic/inflammatory effect on Leydig cells in the testes associated with a decrease in Leydig cell number [285].

Beyond viral infections, the literature also suggests that chronic bacterial infections can drive to male infertility by negatively affecting spermatogenesis and testosterone synthesis by enhancing tissue damage, inflammation and oxidative stress [286,287]. Acutely, certain bacterial infections such as the uropathogenic *Escherichia coli* or *Staphylococus aureus* can be triggered by testosterone through direct and indirect mechanisms [288,289], suggesting that the role of testosterone on bacterial infections is multidirectional and context-dependent. In a similar way, parasitic infections caused by *Plasmodium* spp. *Trichomonas vaginalis*, *Trypanosoma brucei* has also been associated with reduced testosterone levels in previous studies [290]. Collectively, compelling evidence seems to support that chronic viral, bacterial or parasitic infections are important factors associated with testosterone decline in men, with a special mention of the long-lasting effects studied caused by SARS-CoV-2 in a significant proportion of men.

Despite not being considered an infection, gut dysbiosis (mainly represented by reduced diversity of different bacterial phyla and an overgrowth of certain species) is a chronic condition related to lifestyle and other factors that may contribute to the observed secular decrease in testosterone levels. For instance, recent studies have demonstrated that the presence of certain species like *Pseudomonas nitroreducens* can contribute to hyperlipidemia in humans by degrading testosterone through the enzyme 3/17β-hydroxysteroid dehydrogenase (3/17β-HSD) [291]. Another study drew similar conclusions in patients with MDD, reporting an overgrowth of *Mycobacterium neoaurum*, responsible for degrading testosterone by the 3β-HSD enzyme. Beyond individual bacterial species capable of directly degrading androgens, broader gut microbiota alterations have been associated with reduced circulating testosterone levels. Metagenomic and hormone-association studies have identified microbial signatures predictive of lower testosterone, characterized by an enrichment of Firmicutes (notably Ruminococcaceae and Lachnospiraceae) and taxa such as Prevotellaceae, together with a relative depletion of bacteria involved in steroid metabolism and metabolic homeostasis, including Akkermansia and Bifidobacterium [292]. These associations have been observed across sexes and are markedly attenuated by obesity, suggesting that testosterone-related dysbiosis is both compositional and functional, rather than restricted to isolated androgen-degrading species [292]. Therefore, gut dysbiosis should be considered as a critical mechanism potentially related to the secular decrease in testosterone, although this area of knowledge requires further and deep research.

##### Urbanization and Air Pollutants

Finally, air and environmental pollutants—specially particulate matter (PM2.5 and PM10), sulfur dioxide (SO_2_), nitrogen oxides (NO_2_), ozone, and heavy metals—have been strongly associated with reduced testosterone-related reproductive parameters, including sperm concentration, motility, morphology, and DNA integrity, as described by recent reports [193]. These effects are likely mediated by several mechanisms: (1) oxidative stress, which increases reactive oxygen species (ROS) and damages sperm DNA; (2) endocrine disruption, via polycyclic aromatic hydrocarbons (PAHs) and heavy metals with estrogenic and antiandrogenic properties that impair gonadal steroidogenesis; and (3) DNA damage and epigenetic alterations, such as DNA adduct formation and methylation changes [193]. More specifically, cadmium (Cd), lead (Pb), mercury (Hg), aluminum (Al), and arsenic (As) impair testosterone production and male reproductive function through multiple mechanisms, including downregulation of key steroidogenic enzymes (3β-HSD, 17β-HSD, cytochrome P450 enzymes, StAR), damage to Leydig and interstitial cells, reduction in androgen receptors, and disruption of intracellular signaling pathways such as cAMP [293,294,295,296,297]. Additionally, these metals induce oxidative and peroxidative stress, sperm DNA damage, and decreased sperm quality, collectively reducing circulating testosterone levels and fertility. In parallel, PM2.5 exposure inhibits testosterone synthesis and reduces testosterone levels by triggering ferroptosis through the SIRT1/HIF-1α signaling pathway [298]. PM2.5 causes apparent structural impairment of seminiferous tubules, Leydig cell vacuolization, decline in serum testosterone, and sperm quality reduction [298]. Urbanization processes compound these effects by concentrating populations in environments with higher pollution exposure, reduced physical activity opportunities, and increased psychosocial stressors, while simultaneously altering dietary patterns toward processed foods that further disrupt hormonal homeostasis. 

**Figure 3:**
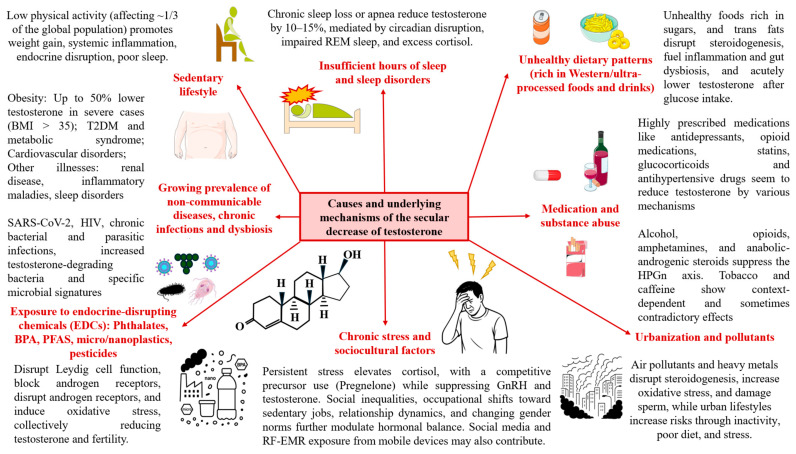
Causes and mechanisms underlying the secular decline in testosterone levels. Contributing factors include obesity (body mass index [BMI] > 35–40 reduces testosterone by up to 50%) through suppression of the hypothalamic–pituitary–gonadal (HPGn) axis, chronic inflammation, insulin resistance, and increased aromatization. Sedentary lifestyle, poor dietary patterns (high in sugars, trans fats, ultra-processed foods), insufficient sleep, chronic stress, and sociocultural influences further exacerbate reductions in testosterone. Environmental and lifestyle contributors include exposure to endocrine-disrupting chemicals (EDCs; e.g., phthalates, bisphenol A [BPA], per- and polyfluoroalkyl substances [PFAS], pesticides, micro/nanoplastics), alcohol, opioids, amphetamines, anabolic-androgenic steroids, and chronic exposure to air pollutants and radiofrequency electromagnetic radiation (RF-EMR). Collectively, these disrupt Leydig cell function, androgen receptor signaling, and increase oxidative stress, reducing fertility and testosterone levels.

Overall, in Figure 3, the potential causes of the secular testosterone decline in modern times are summarized.

#### 4.2.2. Consequences

The age-independent nature of the decline means that younger men today are experiencing testosterone levels that would have been considered pathologically low in previous generations. This trend has resulted in an increasing proportion of men meeting biochemical criteria for hypogonadism across all age groups. The continuation of this trend could have significant implications for population health, fertility rates, and healthcare costs in the coming decades, especially for youths, in which testosterone deficiency nowadays has a prevalence of 20% among adolescent and young adult males [165]. These data could be even higher, as—despite the fact that the cutoff for diagnosing hypogonadism is 300 ng/dL—authors like Zhu et al. [56] claimed that these values need to be revisited, and cutoffs for low testosterone levels should be 409, 413, 359, 352, and 350 ng/dL for 20–24, 25–29, 30–34, 35–40, and 40–44 years old, respectively.

Among the main consequences of low testosterone levels, fertility concerns represent one of the major issues to address in public health. The fertility implications are particularly concerning, as testosterone plays essential roles in spermatogenesis and overall reproductive function. Studies have reported concurrent declines in sperm counts and increases in testicular cancer rates, suggesting a broader pattern of male reproductive health deterioration [299,300]. More specifically, a systematic review and meta-regression analysis [301] found that between 1973 and 2018, sperm count fell by more than 51%, with a notable acceleration after 2000, reaching an annual decrease of 2.64% per year. Low testosterone levels are associated with decreased sexual desire, which is the most important correlate of male hypogonadism, while the relationship between testosterone deficiency and erectile dysfunction is multifaceted, with testosterone playing essential roles in nitric oxide production, adenosine signaling, calcium sensitization via RhoA-ROCK pathway and even penile smooth muscle differentiation.

Apart from this, lower testosterone levels are associated with increased risks of various diseases and a recent work found that lower levels of testosterone were related to higher mortality for the majority of disease categories in either an age-dependent or age-independent fashion [302]. However, the causal role of testosterone decline in mortality is still unclear and as aforementioned multiple diseases promote testosterone decrease, which in turn exacerbates their progression.

Apart from the aforementioned metabolic disorders, neurodegenerative disorders represent an important consequence of testosterone decline, with epidemiological studies demonstrating associations between lower testosterone concentrations and higher prevalence and incidence of cognitive decline, dementia, and Alzheimer’s disease in middle-aged and older men [31]. Men with testosterone concentrations in the lowest quintile show higher incidence of dementia and Alzheimer’s disease [303], with mechanistic studies indicating that testosterone may prevent β-amyloid accumulation in hippocampus, subiculum, and amygdala regions, potentially providing protective effects against dementia pathology [31]. Certain observational studies of men with prostate cancer treated with androgen deprivation therapy have also demonstrated higher risk of cognitive impairment and dementia [304]; however, further studies are warranted to establish a possible causal role of this therapeutic approach, with notable benefits for these patients.

Autoimmune disease susceptibility represents another critical consequence, as hypogonadism is associated with a 33% increased risk of developing rheumatic autoimmune diseases, including a 31% higher risk of rheumatoid arthritis and 58% elevated risk of lupus [305]. Sleep architecture and circadian rhythm disruptions constitute additional consequences of testosterone decline, with low testosterone levels promoting reduced sleep efficiency, increased nocturnal awakenings, less time in slow-wave sleep, and impaired sleep quality that requires testosterone replacement therapy for improvement [306]. Sarcopenia and frailty progression accelerate with testosterone deficiency, as the hormone directly interacts with androgen receptors in myonuclei and satellite cells, with low testosterone levels strongly associated with decreased muscle strength, reduced body lean mass, decreased bone mineral density, and increased morbidity and mortality risks among aging populations [307]. Bone health and fracture risk represent critical consequences of testosterone deficiency, with men having testosterone levels in the lowest quartile showing two-fold higher risk of subsequent fractures compared to those with normal or high levels, independent of age and weight [308]. Low testosterone levels, particularly reduced free testosterone and bioavailable testosterone, are independent predictors for osteoporotic hip fractures in elderly men, with hypogonadism accounting between 16% and 30% of male osteoporosis cases [309].

The socioeconomic impact of testosterone deficiency is starting to be elucidated by different studies. Moskovic et al. [310] reported that over a 20-year timeframe, testosterone deficiency is estimated to contribute to the onset of roughly 1.3 million new CVD cases, 1.1 million new cases of T2DM and more than 600,000 osteoporosis-related fractures (ORFs) in the United States. In the first year alone, the economic burden of these conditions was estimated at approximately $8.4 billion and when projected over the full 20 years, testosterone deficiency could be directly responsible for $190–$525 billion in U.S. healthcare costs, adjusted for inflation.

Understanding the psychosocial role of testosterone has important implications for mental health and social functioning, as disruptions in this regulatory system contribute to various psychological disorders and maladaptive social behaviors [27,233]. Chronic stress-induced testosterone suppression has been linked to depression, reduced motivation, and impaired social confidence, while excessive testosterone responses to minor social challenges may contribute to aggression and antisocial behavior patterns [27,124]. Research indicates that depressive symptoms are reported in 35–50% of men with hypogonadism, with testosterone serving as a biomarker for depression risk [311].

**Figure 4:**
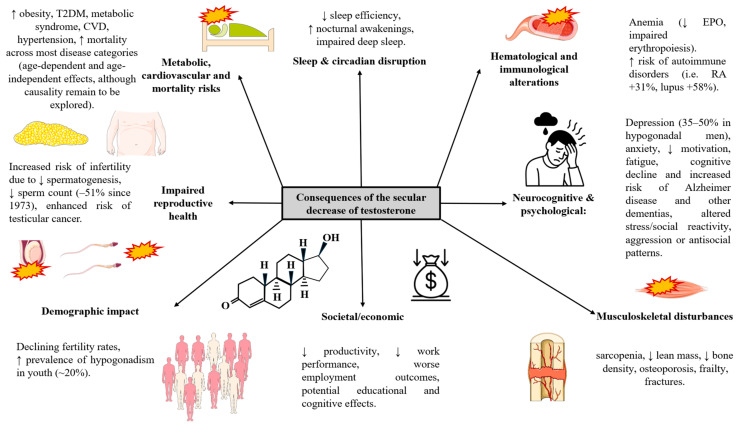
Consequences of the secular decrease in testosterone levels. Reduced testosterone impairs reproductive health, contributing to decreased spermatogenesis, declining sperm counts (–51% since 1973), increased testicular cancer risk, and higher prevalence of hypogonadism among young men (~20%). Metabolic and cardiovascular consequences include increased obesity, type 2 diabetes mellitus (T2DM), metabolic syndrome, hypertension, cardiovascular disease (CVD), and overall mortality. Hematological and immune alterations include anemia (↓ erythropoietin [EPO], impaired erythropoiesis) and increased autoimmune disease risk (e.g., rheumatoid arthritis [RA] +31%, lupus +58%). Psychological and neurocognitive effects encompass depression (35–50% in hypogonadal men), anxiety, cognitive decline, fatigue, altered stress reactivity, aggression, and antisocial behaviors. Musculoskeletal consequences include sarcopenia, decreased lean mass, reduced bone density, osteoporosis, and frailty. Sleep and circadian rhythm disturbances further compound these outcomes, with broad societal and economic repercussions (reduced productivity, work performance, and educational/cognitive outcomes). Note: Arrows indicate direction of change; (↑) denotes an increase and (↓) denotes a decrease.

Economic and workplace productivity implications emerge as significant consequences of the testosterone decline, with testosterone deficiency symptoms including mood disorders, low energy, poor concentration, memory loss, and poor sleep quality directly threatening workplace performance and productivity [312,313]. Studies demonstrate that higher testosterone levels predict better employment outcomes, with unemployed men having significantly lower testosterone and those with higher testosterone levels showing reduced risk of remaining unemployed [314]. Educational and academic performance impacts may emerge from testosterone-related cognitive changes, with studies indicating that while testosterone itself does not directly determine academic achievement, hormonal imbalances can affect focus, concentration, and cognitive processing abilities that influence learning outcomes [315]. However, Harrison et al. [316] claimed that Mendelian randomization analyses provided little evidence for a causal effect of circulating testosterone with socioeconomic position, health or other variables. These observations reflect that indeed, testosterone itself cannot determine alone such complex factors, but contribute in a broader sense to different results according to the context. Howsoever, potential consequences of the secular testosterone decline are collected in Figure 4.

### 4.3. Limitations of the Current Evidence Supporting Testosterone Decline and Future Research Directions

Although a growing body of literature reports a secular decline in circulating testosterone levels, the strength, magnitude, and clinical relevance of this phenomenon require cautious interpretation. Substantial methodological heterogeneity, unresolved confounding, and inherent statistical limitations collectively challenge the notion of a uniform, population-wide testosterone crisis. The principal limitations of this literature can be grouped into analytical, design-related, confounding, and interpretative domains.

(1) Analytical variability and assay-related limitations: One of the most important methodological concerns arises from inter-assay variability across decades. Studies conducted from the 1970s through the early 2000s predominantly relied on immunoassays (radioimmunoassay, enzyme immunoassay, fluorescence immunoassay), whereas liquid chromatography–tandem mass spectrometry (LC-MS/MS), now considered the gold standard for steroid quantification, was only widely adopted after approximately 2005. As a result, many secular trend analyses inadvertently combine data generated using fundamentally different analytical platforms, potentially introducing systematic bias. Although a limited number of large cohorts attempted to minimize this artifact through rigorous standardization, most earlier studies lacked harmonization across assay generations. Even when statistical recalibration is applied, residual measurement error may persist, particularly at lower testosterone concentrations where immunoassays perform poorly. This methodological evolution complicates longitudinal comparisons and may partially account for the magnitude of the reported decline.

(2) Specimen storage and sample integrity: Heterogeneity in specimen storage conditions represents an additional, frequently underestimated source of bias. Long-term storage of serum samples—sometimes exceeding two decades—has been associated with evaporation and concentration artifacts. For example, elevated sodium concentrations observed in long-stored samples indicate volume loss, necessitating correction factors applied at the cohort level. However, such adjustments cannot fully account for individual-level variability, introducing uncertainty into trend estimates. These limitations raise concerns regarding the comparability of historical and contemporary testosterone measurements.

(3) Study design constraints and temporal inference: A substantial proportion of the secular decline literature is based on cross-sectional designs or longitudinal cohorts with relatively short follow-up periods. These designs limit causal inference and hinder the ability to disentangle true temporal changes from cohort effects. Even in longitudinal analyses, attrition and survivor bias pose significant challenges, as healthier individuals are more likely to remain in follow-up, potentially exaggerating age-related declines. Age–period–cohort (APC) modeling further complicates interpretation due to its inherent statistical non-identifiability: linear trends in testosterone cannot be conclusively attributed to period effects (environmental or societal changes) versus generational cohort differences. Notably, longitudinal studies often report steeper annual declines than cross-sectional analyses, a discrepancy consistent with selective survival rather than true population-wide hormonal deterioration.

(4) Inadequate control of confounding variables: Many studies fail to comprehensively adjust for critical confounders known to influence testosterone levels. Chronic diseases, medication use (e.g., glucocorticoids, opioids, antidepressants), and comorbid metabolic conditions are inconsistently recorded, with detailed adjustment largely confined to a few well-characterized cohorts. Lifestyle factors—including physical activity intensity, sleep duration and quality, alcohol consumption, and dietary patterns—are rarely standardized across historical datasets. Moreover, occupational and environmental exposures represent a major unresolved confounder. Exposure to EDCs, heavy metals (lead, cadmium, mercury), and air pollutants such as fine particulate matter (PM2.5) temporally overlaps with the reported decline but was not systematically measured in mid-20th-century cohorts. The absence of these variables limits causal attribution and raises the possibility that observed trends reflect unmeasured environmental shifts rather than intrinsic endocrine aging.

(5) Biological interpretation and clinical relevance: Even where a secular decline in total testosterone is convincingly demonstrated, its clinical significance remains debated. Several large studies report relatively stable free or bioavailable testosterone despite reductions in total testosterone, suggesting changes in binding dynamics—particularly involving sex hormone-binding globulin—rather than a true decline in androgenic activity. Furthermore, substantial interindividual variability exists in symptom expression at equivalent testosterone concentrations, influenced by androgen receptor polymorphisms, 5-α-reductase activity, and genetic variation in SHBG. These biological modifiers are not captured by simple hormone measurements, limiting the ability to translate population-level declines into clinically meaningful conclusions.

Collectively, these limitations underscore the need for more rigorous methodological approaches. Future studies should prioritize standardized LC-MS/MS assays, repeated within-individual measurements to reduce variability, comprehensive assessment of lifestyle, medication, and environmental exposures, and a greater focus on free or bioavailable testosterone rather than total concentrations alone. Such approaches may ultimately reframe the secular decline narrative from a generalized hormonal crisis to a more nuanced alteration in testosterone regulation and binding dynamics with preserved biological activity.

## 5. Potential Interventions Directed to Enhance Testosterone Levels

After exploring the evidence, causes, and consequences of the secular decline in testosterone, this section examines potential interventions to increase testosterone levels in the general population. It is important to first recognize the influence of non-modifiable factors such as sex, age, and genetics. While sex differences were discussed earlier, age and genetics remain crucial determinants of testosterone regulation.

Age is one of the strongest and inevitable factors. Testosterone begins to decline around 35–40 years, with total T decreasing ~1.6% annually, and free/bioavailable T at 2–3% due to rising SHBG levels [317,318]. Mechanisms include both primary testicular dysfunction (Leydig cell loss and reduced LH responsiveness: ~44% fewer Leydig cells in older vs. younger men) and secondary hypothalamic–pituitary changes (reduced GnRH secretion, altered pulsatility) [318,319,320,321,322]. While primary hypogonadism in older men is generally irreversible, secondary forms are more common in younger men and may be modifiable [318]. The clinical significance of this age-related decline is substantial, with approximately 20% of men over 60 and 50% of men over 80 having testosterone levels below the normal range for young adults [320,323].

Genetics explains ~40–70% of serum testosterone variation, as shown in twin studies [6,324,325,326]. The genetic architecture of testosterone regulation is highly complex and sex-specific, with almost no correlation between men and women [327,328]. Recent large-scale genome-wide association studies (GWAS) have identified over 60 genetic variants associated with testosterone levels, including variants in genes involved in steroidogenesis (*GRAMD1B*), hormone regulation (*JMJD1C*, *LIN28B*), and androgen metabolism [6,329]. The most significant genetic factors involve the SHBG gene, where polymorphisms such as rs12150660 and rs6258 can dramatically affect testosterone concentrations [324]. Men carrying three or more risk alleles of these variants have a 6.5-fold higher risk of having low testosterone compared to those with no risk alleles Additionally, the rs6258 polymorphism affects SHBG’s binding affinity for testosterone, directly influencing free testosterone levels [324]. Other notable loci include FAM9B (rs5934505) on the X chromosome and AR CAG repeat polymorphisms that modulate androgen sensitivity [330,331,332]. These genetic factors create substantial individual variation in testosterone levels and response to hormonal interventions, highlighting the importance of personalized approaches to testosterone management.

However, beyond these non-modifiable factors, the secular testosterone decline is strongly linked to modifiable lifestyle and environmental factors. Evidence supports non-pharmacological interventions such as weight loss, diet, physical activity, stress reduction, sleep optimization, and sunlight exposure as key strategies to naturally improve testosterone. Pharmacological options, including testosterone replacement therapy (TRT), remain indicated only for men with clinically confirmed hypogonadism, while certain nutritional supplements may provide benefit in specific contexts. Apart from this, minimizing the exposure to EDCs, UPFDs, substances like alcohol and other drugs and air pollutants should also be considered as critical measures to enhance testosterone levels in men.

### 5.1. Non-Pharmacological Interventions

#### 5.1.1. Weight Loss

Weight loss represents one of the most powerful non-pharmacological interventions for enhancing testosterone levels, particularly in overweight and obese men. Research demonstrates that losing weight through a balanced diet and regular exercise can boost testosterone production [34,181]. The relationship is dose-dependent, with greater weight loss producing more substantial improvements in testosterone levels. In one study, weight loss of 15% or more can lead to significant increases in both total and free testosterone, likely through reactivation of the HPT axis [333]. Modest reductions in weight (<15%) are linked to only small rises in total testosterone, largely due to increases in SHBG without changes in free testosterone. This improvement occurs regardless of the method used to achieve weight loss, whether through dietary intervention, exercise, or bariatric surgery.

#### 5.1.2. Physical Activity

Exercise represents a potential non-pharmacological strategy to stimulate testosterone production. The acute hormonal response depends on training volume, the amount of muscle mass involved, and inter-set recovery times. Importantly, individual variability in hormonal responses must be considered, with factors such as age, baseline fitness, nutritional status, and circadian rhythms influencing the testosterone response to exercise. In general terms, engaging in physical activity, particularly at moderate to high intensities, can acutely increase testosterone levels. This effect is most noticeable immediately after exercise and within the first 30 min, according to a meta-analysis conducted by D’Andrea et al. [334]. Research shows that resistance exercise—especially when involving large muscle groups, high volume, moderate-to-high intensity, and short rest intervals—can acutely elevate serum testosterone, though the effect is smaller in older or obese men [335,336]. Protocols with moderate intensity (i.e., around 70% 1RM), higher total volume, and short rest periods consistently produce the most robust testosterone increases [337]. Likewise, heavy resistance exercise involving large muscle groups causes immediate increases in testosterone that can persist for 48 h post-exercise, with free-weight exercises producing greater testosterone responses than machine-based exercises due to greater overall muscle mass involvement [335]. High-intensity interval training (HIIT) shows promise for testosterone enhancement, particularly in men, where interval protocols can produce significant testosterone increases [338,339,340].

Endurance exercise presents a more complex relationship with testosterone. When compared to resistance training, endurance training did not report the same benefits to increase testosterone levels in children, adolescents and adults [341,342,343]. Even, chronic endurance trained men (commonly distance running, cycling, race walking, and triathlon training) tend to present low resting testosterone—an observation designed as exercise-hypogonadal male condition—probably due to a dysfunction/disruption of the HPGn axis related to energy availability or a readjustment of the axis for proper physiological functions [344]. Conversely, in elder people, HIIT and endurance training showed more potential to increase baseline testosterone levels than resistance training, according to a recent meta-analysis [345].

However, evidence for long-term increases in resting testosterone from exercise is limited and inconsistent, with potential benefits seen in older and overweight individuals, though it is unclear whether these effects are due to exercise itself or associated weight loss [335]. Additionally, one meta-analysis [346] found that exercise training, regardless of type, age, or body composition, does not significantly alter resting testosterone levels in insufficiently active but otherwise healthy men. This suggests that while exercise has many health benefits, it is unlikely to produce by itself meaningful long-term changes in basal testosterone in this population and a broader context in which additional lifestyle and environmental interventions are required. Further research is needed to establish dose–response relationships, optimal exercise modalities for older men, and whether exercise interventions can fully counteract the secular decline in testosterone or merely mitigate its health consequences

#### 5.1.3. Nutritional Interventions and Dietary Patterns

Nutritional interventions play a fundamental role in supporting testosterone production through both macronutrient and micronutrient optimization. Adequate intake of healthy fats, proteins, carbohydrates, and micronutrients is essential to sustain normal testosterone synthesis and action. In terms of dietary components, fat intake is essential for testosterone production, as cholesterol serves as the foundational substrate for steroid hormone synthesis [35]. Studies demonstrate that men consuming 20% fat diets have significantly lower testosterone levels compared to those on 40% fat diets [347,348]. The type of fat is also a factor of great relevance. Higher intake of monounsaturated (MUFA) and saturated fatty acids (SFA) appears to support testosterone production, while higher intake of polyunsaturated fatty acids (PUFA), particularly omega-6, has been associated with lower testosterone levels in some contexts, potentially by increasing oxidative stress and disrupting testicular function [347]. Consumption of omega-3 PUFAs from fish oil or supplementation is associated with enhanced testosterone production as well [349,350].

Protein intake and carbohydrates can also influence testosterone levels. Regarding carbohydrates, one study has reported enhanced testosterone production in 7 healthy men after a high carbohydrate diet along with reduced cortisol levels, even when comparing with a high protein diet [351]. In a meta-analysis, low-carbohydrate (≤35% of total calories) and very high protein diet (>35% of total calories) appears to cause a large decrease in resting total testosterone (∼5.23 nmol/L) [352]. However, in another study, the author argues that these results must be extrapolated to very-high protein intakes (>3.4 g/kg/day), whereas high- and moderate-protein diets (1.25–3.4 g/kg/day) do not seem to negatively affect testosterone levels [353]. Additionally, the recommended dietary allowance for protein established by the Institute of Medicine at 0.8 g/kg/d for the entire adult population seem to be sufficient for maintaining the anabolic effects of testosterone even in old adults [354]. These findings were further supported in other studies demonstrating that diets low in proteins (0.5 g/kg/d) show negative correlations with testosterone levels in comparison to men reaching the minimum recommended (0.9 g/kg) [355]. Taken together, these findings suggest that adequate carbohydrate availability—ideally adapted to physical activity demands—and sufficient, but not excessive, protein intake are key determinants of optimal testosterone production.

Micronutrients (vitamins and minerals) are equally crucial factors implicated in testosterone synthesis. The literature recognizes the relevance in testosterone production of zinc (found in oysters, red meat, nuts), magnesium (leafy dark greens, nuts, seeds), vitamins from the B complex (particularly B6 and B12) and vitamin D [356,357,358,359,360]. In addition, several trace elements and environmental metals have been associated with testosterone regulation including cadmium, copper and lead, directly associated with testosterone levels and manganese, molybdenum and selenium, all three linked to reduced testosterone [294].

Regarding specific dietary interventions, the literature reveals substantial heterogeneity in outcomes across different dietary patterns and testosterone concentrations, largely confounded by the concurrent effects of weight loss, baseline metabolic status, and overall dietary composition. Consequently, interpretation of dietary effects on testosterone requires careful consideration of individual metabolic and hormonal context.

Ketogenic diets (KD), is a type of dietary pattern characterized by severe carbohydrate restriction and high fat intake, inducing a metabolic state resembling fasting. Among the proposed mechanisms by which KDs (with carbohydrates, fats, and proteins provide approximately 13%, 44%, and 43% of the total energy) can enhance testosterone are (1) By increasing the cholesterol bioavailability in the body; (2) by exerting anti-inflammatory effects; (3) Through reducing appetite and promoting weight loss and (4) By the signaling role of ketone bodies on the hydroxycarboxylic receptor 2 (HCAR2) in the testes, although its relationship with testosterone production should be still deciphered [361].

In humans, the benefits of these interventions have been demonstrated in various studies. In a systematic review and meta-analysis including 230 patients from 7 studies (with only 3 studies enrolling overweight/obese men) KD leaded to elevated serum testosterone, with more benefits observed in those who presented greater weight loss [362]. Collectively, these findings suggest that the apparent testosterone-raising effects of KDs are largely mediated by weight loss and improvements in metabolic health rather than by direct stimulation of gonadal steroidogenesis. Accordingly, the benefits of KDs appear particularly pronounced in overweight and obese men, especially when very low-calorie ketogenic diets (VLCKD) are employed. After an 8-week intervention of VLCKD in 40 obese men, total testosterone seemed to increase along with serum vitamin D and LH [363], accompanied by significant weight loss and improvements in glucose homeostasis and lipid profile.

In another study conducted in 22 obese men over 28 days [364], VLCKD seemed to produce rapid elevations in total testosterone, with greater responses in hypogonadal subjects; however, these gains were accompanied by concurrent increases in SHBG, with calculated free testosterone remaining unchanged. This SHBG-mediated dissociation between total and free testosterone, together with evidence that three-week KD exposure reduces free testosterone despite elevating total testosterone in obese individuals [365], raises important questions regarding the clinical significance of total testosterone elevation independent of bioavailable hormone changes. Finally, apart from overweight/obese subjects, normocaloric KD can also lead to increased testosterone levels and benefit athletes with normal weight in certain contexts [362]. For instance, various works have shown that combined interventions of KD with resistance training lead to increased levels of testosterone when compared to other dietary interventions [366,367], with other studies showing negative effects on testosterone levels on experienced natural bodybuilders [368] or neutral influences on cyclists and cross-fit trained athletes [369,370]. These divergent findings further underscore the importance of individual context when interpreting dietary interventions.

Mediterranean diet (MD) is a pivotal dietary intervention characterized by high intakes of plant-based foods (vegetables, fruits, nuts, whole grains, extra virgin olive oil -EVOO) and moderate intake of fish and seafood, white meat, eggs, dairy products and in some cases fermented alcohol (preferably in form of red wine) [371,372]. On the contrary, consumption of red meat, processed meats, and foods rich in sugars and unhealthy fats should be small in both quantity and frequency. MUFAs and bioactive compounds from EVOO (i.e., oleocanthal) and different polyphenols found in plant-based sources (i.e., resveratrol and quercetin) may indirectly support testosterone homeostasis by reducing oxidative stress, inflammation, and vascular dysfunction [361].

Nevertheless, evidence regarding the direct effects of MD on testosterone levels remains mixed. Some studies have reported no significant differences or even modest reductions in testosterone among individuals with high MD adherence compared with less restrictive diets [373,374]. On the other hand, other studies have found favorable results from this dietary intervention. Chrysohoou et al. [375] found that MD improves erectile function, aortic health (less stiffness), and potentially testosterone in 667 older men, by reducing oxidative stress and inflammation, boosting antioxidants, and supporting vascular health crucial for sexual capacity. In a similar sense, Corsetti et al. observed that a low-carb organic MD increased testosterone levels and reduced sperm DNA fragmentation by a 35% in 50 subfertile men [376]. These discrepancies likely reflect heterogeneity in how MD is operationalized across studies, with some definitions emphasizing high carbohydrate and low fat intake—misaligned with traditional Mediterranean dietary principles—whereas others prioritize fat quality over quantity.

Vegetarian and vegan diets (VDs) present a nuanced hormonal profile distinct from the macronutrient-centric dietary patterns previously discussed. The largest cross-sectional study to date, examining 233 vegan men, 226 meat-eaters, and 237 vegetarians, found that vegan men demonstrated higher total testosterone concentrations compared to both meat-eaters and vegetarians; however, this elevation was accompanied by a concurrent and proportional increase in SHBG, resulting in no significant difference in calculated free testosterone, androstanediol glucuronide, or luteinizing hormone between dietary groups [377]. Thus, the apparent elevation in total testosterone among vegans did not translate into increased bioavailable androgen levels. More recent analyses using standardized plant-based diet indices (PDI) in NHANES data likewise found no association between plant-based diet adherence and serum testosterone levels [378].

The heterogeneity in cross-sectional findings appears attributable to confounding of plant-based diet status with concurrent low-fat diet adoption (which independently suppresses testosterone),¡ rather than to plant-based composition per se, although the literature also suggest that VDs can be deficient in the bioavailability of certain micronutrients (particularly vitamin B_12_, vitamin D, zinc, calcium and selenium), and PUFAs of the omega-3 family, specially eicosapentaenoic (EPA) and docosahexaenoic (DHA) acids, which may impair testosterone synthesis [379,380]. Notably, concerns regarding phytoestrogen-mediated testosterone suppression from soy consumption have been thoroughly refuted by meta-analysis of 32 randomized controlled trials demonstrating no significant effects of soy protein or isoflavone intake on total testosterone, free testosterone, estradiol, estrone, or SHBG in men [381].

Additional dietary approaches such as intermittent fasting (IF) also appear to modulate testosterone, although evidence remains less robust. The relationship between IF and testosterone shows mixed results across studies, with some research demonstrating potential benefits in obese men through improved body composition and reduced inflammation, while other studies indicate decreased testosterone levels in lean, physically active men (without evidence of affecting muscle mass and muscular strength and showing improvements in different health variables) [382,383,384]. The effects of IF on testosterone appear to depend on individual factors including baseline body composition, fasting duration, and metabolic status, with short-term fasting potentially increasing LH by 67% and testosterone by 180% in non-obese men in one study [385], while prolonged fasting (3 days) may reduce testosterone levels by up to 35% [386]. Overall, the hormonal effects of IF appear to depend on baseline body composition, fasting duration, and metabolic resilience.

In synthesis, dietary optimization for testosterone appears less dependent on isolated macronutrient ratios than on: (1) maintenance of adequate energy availability in lean individuals; (2) avoidance of extreme caloric restriction in normal-weight men; (3) prioritization of micronutrient-dense whole foods over refined and ultra-processed products; (4) emphasis on dietary fat quality (MUFAs, SFAs, and balanced omega-6:omega-3 ratios) rather than fat quantity alone; (5) ensuring sufficient intake of healthy fats, proteins (likely between 0.8 and 0.9 to 3.4 g/kg/day), and carbohydrates adapted to physical activity levels; and (6) weight optimization toward a normal BMI in overweight populations, where metabolic restoration may represent the primary mechanism underlying testosterone improvements.

#### 5.1.4. Gut Microbiota Modulators

Clinical interventions targeting gut microbiota also represent an important nutritional strategy directed to increase testosterone levels. A very recent work [387] has demonstrated that higher dietary live microbe intake from fermented foods is associated with a reduced risk of testosterone deficiency (with an OR of 0.71 for the high intake group) in an US cohort with 4034 male participants. However, the use of other gut microbiota-based approaches to modulate testosterone levels have yielded mixed results in human studies. A recent 12-week randomized, double-blind, placebo-controlled trial investigating Limosilactobacillus reuteri ATCC PTA 6475 supplementation in healthy men aged 55–65 years found no significant effect on testosterone levels despite previous animal studies showing promising results [388]. Studies investigating probiotic mixtures containing multiple Lactobacillus strains for male reproductive health are ongoing, with preliminary research suggesting safety and tolerability in healthy volunteers [389], although efficacy data on testosterone levels remain limited. Synbiotic interventions combining probiotics with prebiotics have also shown promising results, particularly in animal models where a synbiotic combination of Lactobacillus gasseri 505 with Cudrania tricuspidata leaf extract successfully prevented chronic stress-induced testosterone suppression by upregulating steroidogenic acute regulatory protein (StAR) and luteinizing hormone receptor (Lhr) expression while normalizing testicular development-related genes [390]. Thus, although dietary live microbe intake from fermented foods shows correlational benefits for testosterone levels, controlled trials using specific probiotic supplements have not consistently demonstrated testosterone-enhancing effects in healthy men, suggesting that the relationship between gut microbiota modulation and testosterone regulation may be more complex than initially hypothesized.

#### 5.1.5. Sleep Optimization

Evidence from multiple controlled studies demonstrates that sleep interventions can effectively improve testosterone levels in men, with the most compelling data coming from sleep restriction trials that reveal dramatic improvements when normal sleep is restored. A landmark study by Leproult and Van Cauter [224] found that just one week of sleep restriction to 5 h per night reduced daytime testosterone levels by 10–15% in healthy young men, with the most pronounced effects occurring between 2 PM and 10 PM. When participants returned to normal sleep (8 h), testosterone levels recovered to baseline within days, demonstrating the reversible nature of sleep-induced testosterone suppression. More dramatic evidence comes from military studies where a single night of total sleep deprivation during Ranger training reduced testosterone by 25–30%, with morning testosterone levels dropping to roughly 90% below expected values at the time of the natural testosterone peak [391]. Community-based research supports these findings, showing that older men with longer sleep duration (≥9.5 h) had significantly higher testosterone levels compared to those with short sleep duration (<6.5 h), with each additional hour of sleep associated with a 75 ng/dL increase in testosterone after adjusting for multiple confounding factors [392]. In more detail, Auyeung et al. [393] found that sleep duration up to 9.9 h increased testosterone levels, after which it decreased, giving rise to an inverted U-shaped relationship.

While sleep extension studies have shown mixed results for testosterone enhancement beyond normal duration, the consistent finding across all research is that maintaining 7 to 9 h of quality sleep is essential for optimal testosterone production. Indeed, testosterone production is intimately linked to sleep, with most testosterone secretion occurring during sleep, particularly during REM sleep phases [223,225]. The relationship is both circadian rhythm-dependent and sleep-dependent, requiring at least 3 h of uninterrupted sleep with normal architecture for optimal testosterone production [225]. Testosterone secretion exhibits distinct circadian rhythmicity with peak levels occurring between 6:00 and 8:00 a.m. and nadir values in the late afternoon/early evening [394,395,396,397], driven by the suprachiasmatic nucleus (SCN) through regulation of GnRH and LH pulsatile release [398,399]. The circadian pattern is mediated by core clock genes including BMAL1, CLOCK, PER1/2, and CRY1/2, which create transcriptional-translational feedback loops within Leydig cells to control steroidogenic gene expression [400,401]. Specifically, the BMAL1-CLOCK heterodimer binds to E-box sequences in promoter regions of steroidogenic genes like StAR, HSD3B2, and CYP17A1, orchestrating rhythmic testosterone production [400,401]. These chronobiological mechanisms create a complex regulatory network where testosterone serves both as a circadian-controlled hormone and as a modulator of circadian function through androgen receptors located within the SCN core region [402]. Overall, sleep architecture significantly influences testosterone regulation, with increases occurring during the first 3 h of sleep coinciding with REM episodes, though this relationship weakens with advancing age as circadian amplitude diminishes by approximately 10–35% between young and elderly men [403].

#### 5.1.6. Stress Management

Stress management represents another crucial component of testosterone enhancement. Evidence from controlled trials demonstrates that psychotherapeutic interventions, particularly cognitive-behavioral stress management (CBSM) and mindfulness-based approaches, can effectively improve testosterone levels through stress reduction and hormonal regulation mechanisms. A randomized controlled trial by Cruess et al. [404] found that a 10-week group-based cognitive-behavioral stress management intervention significantly increased free testosterone levels in HIV-seropositive men compared to wait-list controls, who actually showed significant decreases in testosterone during the same period. The testosterone improvements were inversely related to reductions in psychological distress, independent of cortisol changes, suggesting that CBSM works through multiple pathways beyond simple stress hormone modulation.

Recent research on mindfulness meditation provides particularly compelling evidence, with a randomized controlled trial showing that seven 20 min sessions of Integrative Body–Mind Training (IBMT) modulated both testosterone and cortisol responses to acute stress more effectively than relaxation training [405]. Participants in the mindfulness group experienced higher testosterone concentrations following an additional practice session immediately after stress exposure, while the relaxation group showed higher cortisol levels at the same point, suggesting that mindfulness meditation can co-regulate the HPA and HPT axes. By reducing perceived stress and cortisol reactivity, these psychological interventions create a more favorable hormonal environment for testosterone synthesis, with the added benefits of improved mood, better sleep quality, and enhanced psychological flexibility that further support optimal endocrine function.

Finally, exposure to nature (i.e., forest bathing or shinrin-yoku) contributes to testosterone optimization through stress hormone modulation, with multiple studies showing that forest immersion significantly reduces cortisol, adrenaline, and noradrenaline levels while enhancing immune function and psychological well-being [406,407]. One of the reported mechanisms by which forest bathing modulate cortisol and other hormones are attributed to natural substances known as phytoncides (volatiles substances from trees like alpha-pinene, limonene, and beta-caryophyllene). However, these substances do not directly increase testosterone but create optimal conditions for hormonal balance by reducing chronic stress and cortisol elevation, along with their modulatory effects on the immune system and inflammation [406,408], which are known suppressors of the HPT axis. Other stress-reduction techniques such as regular physical activity, adequate sleep, and limiting negative thinking patterns can also help maintain optimal hormonal balance.

#### 5.1.7. Sun Exposure

Evidence demonstrates that sun exposure can positively influence testosterone levels through multiple physiological pathways, though the effects vary depending on the intervention type and study population. Firstly, ultraviolet (UV) light from sun exposure is responsible for producing over 80% of the endogenous vitamin D [409]. Vitamin D deficiency has been associated with reduced testosterone levels [410,411]. Importantly, the amount of sun required for maximizing vitamin D synthesis depends on different factors such as the type of skin, the latitude, season of the year, time of day or the use of sun protection factors (SPFs). For instance, a fair-skinned person at midday in June in Boston will maximize their vitamin D photosynthesis in much less than 5 min, whereas if applying a SPF-15 sunscreen for around 20 min would be required to maximize vitamin D synthesis [412]. Further sun exposure will only produce photodamage.

Despite this, existing literature presents conflicting findings regarding the association between vitamin D supplementation and testosterone, with some studies reporting a direct relationship while others show no such correlation [411,413,414]. In a similar sense, vitamin D supplementation combined with sun exposure has demonstrated significant benefits in testosterone levels in specific population such as elite athletes [415] or obese individuals [416]; however, the role of these interventions in the general population remain inconclusive [411].

Other mechanisms apart from vitamin D can explain the positive relationship between sun exposure and testosterone levels. For instance, direct UV light exposure can actually increase testosterone levels by itself, acting by the recently described skin–brain–gonad axis. According to this mechanistic research [417]. UVB exposure activates p53 genes in keratinocyte skin cells, triggering the pituitary gland to release LH and FSH, which subsequently stimulates testosterone production in the testes in mice models. Testosterone levels also follow a seasonal variation, with highest levels in August–October, when UV exposure is highest and nadir levels during winter, with the lowest levels reported in March [418]. Another study also found broader increases in testosterone levels during summer in men from countries with lower UV radiation when compared to those from regions with higher UV radiation, without reporting significant differences in winter [417].

#### 5.1.8. Thermal Interventions

Environmental temperature plays a significant yet often overlooked role in male hormonal and reproductive health. Both heat and cold exposure can influence testosterone production and spermatogenic function through distinct physiological mechanisms. For instance, recent research has suggested that heat stress due to climate warming seems to impair testicular function and testosterone synthesis by damaging Leydig cells, disrupting steroidogenic enzymes, and increasing oxidative stress, with optimal spermatogenesis requiring testicular temperatures 2–6 °C below core body temperature [419]. Chronic exposure to elevated temperatures may abnormally increase aromatase activity, reducing the testosterone-to-estradiol ratio and leading to lower serum and intratesticular testosterone levels, as well as impaired sperm parameters. Thus, avoiding exposure to heat stress can be an effective measure to increase testosterone level. Interestingly, while habitual exposure to heat through sauna use generally shows neutral effects on testosterone, some studies have reported mild benefits among physically active individuals, possibly due to improved stress adaptation and vascular function [420,421,422].

Cold exposure generally decreases testosterone, with studies showing reductions in serum levels and blunted post-exercise hormonal responses, potentially hindering muscle adaptation [423,424,425]. However, evidence remains mixed and context dependent. Some interventions, such as whole-body cryotherapy in athletes, have shown transient or modest increases in testosterone, suggesting that the timing, duration, and intensity of cold exposure may critically determine its endocrine outcomes [426]

Although thermal interventions may not directly elevate testosterone synthesis, they can indirectly support hormonal health through several mechanisms. Both heat- and cold-based therapies have been shown to reduce cortisol levels, enhance metabolic efficiency, lower systemic inflammation, and improve mood regulation [420,427,428]. These effects address key contributors to testosterone decline—obesity, stress, and metabolic dysfunction—making thermal therapies valuable adjuncts in lifestyle-based interventions.

### 5.2. Pharmacological Interventions

#### 5.2.1. Testosterone Replacement Therapy (TRT)

Testosterone replacement therapy (TRT) represents the primary pharmacological intervention for hypogonadism, with multiple formulations demonstrating efficacy in normalizing serum testosterone levels. It is important to highlight that TRT should be only offered to men with clinical hypogonadism, always considering personalized interventions and non-pharmacological approaches. Within TRT, oral, buccal, nasal, subdermal, transdermal, and injectable formulations of testosterone have been developed, each with distinct pharmacokinetics, administration routes, and safety profiles. Oral and buccal preparations are limited by poor bioavailability, gastrointestinal or gum-related side effects, and inconsistent efficacy, while nasal testosterone provides stable serum levels without suppressing spermatogenesis but requires multiple daily doses [429]. However TRT is most commonly administered by intramuscular injection of long-acting testosterone or transdermally via patches or gels, and less commonly via oral Each alternative has their proper advantages and disadvantages, and the choice of administration route is primarily based on patient preference at our institution, and the decision is made collaboratively after a thorough discussion of the pros and cons [430]. For instance, gels and patches allow steady serum T with non-invasive use but risk skin irritation or transfer to other patients, whereas intramuscular injections—especially testosterone undecanoate—offer long-acting, effective replacement but require medical supervision due to specific risks such as erythrocytosis, pulmonary oil micro-embolism (POME), and long washout periods [430,431].

As people with hypogonadism present pathologically low levels of testosterone, the use of TRT has significant benefits on their muscle mass, bone density, body composition, libido and sexual function, mood, erythropoiesis, cognition, quality of life and cardiovascular disease risk [432]. However, the literature also recognizes some potential risks that should be monitored by clinicians. For instance, Layton et al. [433] analyzed 544,115 testosterone initiators and demonstrated that injection initiators had higher hazards of cardiovascular events, hospitalizations and death compared to gel and patch users, likely due to the supraphysiological testosterone spikes associated with injectable formulations. On the other hand, Borst and Yarrow [434] claimed that in older hypogonadal men, intramuscular TRT appears to provide greater musculoskeletal benefits than transdermal TRT, without the same cardiovascular risks, likely because skin—but not muscle—converts more testosterone into DHT. Current evidence, though still limited, suggests that combining intramuscular TRT with finasteride (an inhibitor of the 5-α reductase) may offer the safest and most effective option by enhancing benefits while minimizing risks to the prostate and cardiovascular system [434]. Other studies such as the comprehensive systematic review and individual participant data meta-analysis conducted by Cruickshank et al. [435] evaluated 35 randomized controlled trials (5601 participants) and found that TRT improved quality of life and sexual function in almost all patient subgroups while maintaining cardiovascular safety, with no significant differences in major adverse cardiac events that the benefits from using TRT in hypogonadal men may surpass the deleterious side effects [436,437]. However, these analyses are limited by the relatively short follow-up periods and insufficient power to assess mortality outcomes due to low event rates, and future studies are still warranted to confirm these associations.

The use of testosterone therapy in non-hypogonadal men with normal testosterone levels represents a controversial area that has received significant research attention. Several studies have evaluated testosterone administration in eugonadal men, primarily using supraphysiological doses, produce beneficial effects on body composition and performance enhancement, particularly when combined to resistance training programs [438,439]. Beyond this, there is no evidence of the benefits of testosterone therapy in men with normal baseline testosterone levels in specific areas partially modulated by testosterone levels like sexual libido or mood, and many of the aforementioned risks may appear such as erythrocytosis, cardiovascular complications, testicular atrophy, infertility, gynecomastia, and behavioral changes including increased aggression and manic symptoms [432,440]. Thus, testosterone injections are not recommended for eugonadal men because the practice is not supported by the evidence, and the harms largely overcome the pros.

#### 5.2.2. Supplementation

Certain herbal supplementation has shown promising benefits to increase testosterone levels in men under specific contexts. A systematic review by Smith et al. [38] examining 32 randomized controlled trials of various herbal interventions found that only ashwagandha Withania somnifera) and fenugreek seed extracts (Trigonella foenum-graecum) demonstrated consistent positive effects on testosterone concentrations, with evidence quality limited by small sample sizes, heterogeneous study populations (predominantly young, healthy men), and variation in dosages and extraction methods.

Regarding ashwagandha, a randomized, double-blind, placebo-controlled crossover study by Lopresti et al. [441] demonstrated that 8 weeks of standardized ashwagandha extract (Shoden beads, 600 mg daily delivering 21 mg withanolide glycosides) significantly increased salivary testosterone by 14.7% and DHEA by 18% compared to placebo in overweight men aged 40–70 years with mild fatigue. However, these hormonal improvements did not translate to significant differences in fatigue, vigor, or sexual well-being compared to placebo, and the effects were not sustained after discontinuation, suggesting ongoing supplementation is required to maintain benefits. Similar findings were reported by Chauhan et al. [442] in a study of 50 men with lower sexual desire, where 300 mg of ashwagandha root extract twice daily for 8 weeks significantly increased serum testosterone levels and improved sexual function scores on the derogatis interview for sexual functioning-male (DISF-M) questionnaire compared to placebo. The mechanism appears to involve modulation of the HPA axis and potential upregulation of GnRH activity, as suggested by in vitro and animal studies, rather than direct steroidogenic effects.

For fenugreek extract, a comprehensive meta-analysis by Mansoori et al. [443] including four randomized controlled trials demonstrating significant effects on serum total testosterone levels in males. More recently, Lee-Ødegård et al. [444] conducted a rigorous 12-week randomized, double-blind trial with 95 men aged 40–80 years, showing that Trigozim^R^ fenugreek extract increased plasma total testosterone and free testosterone index by 13.0% and 16.3%, respectively, versus baseline, with saliva testosterone concentrations increasing by 31.1% versus baseline and 37.2% versus placebo. The highest dose (1800 mg) produced the most pronounced effects, with a 19.6% increase in saliva testosterone, suggesting a dose-dependent response mechanism that may involve modulation of 5α-reductase and aromatase enzymes.

Zinc supplementation has demonstrated particularly robust evidence for testosterone enhancement, especially in deficient populations. The seminal work by Prasad et al. [359] established that zinc deficiency directly correlates with hypogonadism, showing that dietary zinc restriction in healthy young men reduced serum testosterone from 39.9 ± 7.1 to 10.6 ± 3.6 nmol/L after 20 weeks, while zinc supplementation (459 μmol/day) in marginally deficient elderly men increased testosterone from 8.3 ± 6.3 to 16.0 ± 4.4 nmol/L over six months. Medicinal doses of zinc (30 mg/day chelated zinc for 1–6 months) can increase total testosterone levels from 180 to 222 ng/dL in deficient men, whereas the highest reported uses of zinc to increase total testosterone 660 mg/day of zinc sulfate, which elevated TT by 190–350 ng/dL, although with gastric side effects that necessitated dose reduction [445]. Lower doses, such as 250 mg/day, also showed significant increases (≈400 ng/dL), surpassing the results of other herbal remedies and vitamin D. The mechanism involves zinc’s essential role in steroidogenic enzyme function and Leydig cell testosterone synthesis, making supplementation most effective in men with documented zinc deficiency or marginal status.

Magnesium supplementation has shown promising results through its effects on sex hormone-binding globulin and testosterone bioavailability. Cinar et al. [360] demonstrated in a controlled trial that 4 weeks of magnesium sulfate supplementation (10 mg/kg/day) significantly increased both free and total testosterone levels in both sedentary subjects and athletes, with greater increases observed in the athletic group. The mechanistic basis for magnesium’s effects was reviewed by Maggio et al. [446], who suggest that magnesium directly modulates testosterone-SHBG binding affinity through nonspecific binding to SHBG, leading to uncompetitive inhibition and subsequent enhancement of bioavailable testosterone. Similarly, magnesium can displace testosterone from its serum albumin binding site in vitro, suggesting that magnesium supplementation during DHEA treatment may enhance Bio-T levels in vivo [447,448].

Other supplements with potential benefits in increasing testosterone levels include boron and DHEA. Boron supplementation has demonstrated rapid effects on testosterone metabolism and inflammatory markers, though the evidence base remains limited. Naghii et al. [449] conducted a small but well-controlled study showing that just one week of boron supplementation (10 mg daily) in healthy men significantly increased free testosterone from 11.83 to 15.18 pg/mL while decreasing estradiol from 42.33 to 25.81 pg/mL, accompanied by substantial reductions in inflammatory biomarkers including a 50% decrease in high-sensitivity C-reactive protein. The mechanism appears to involve boron’s effects on steroid hormone metabolism and SHBG binding, as evidenced by significant increases in the ratios of free testosterone to total testosterone, testosterone to estradiol, and free testosterone to estradiol, suggesting “androgen amplifier effects” that enhance the conversion of bound to free testosterone. However, another study with bodybuilders using 2.5 mg/day of boron for 7 weeks showed no differences in testosterone or other hormonal markers compared to placebo [450]. For DHEA a meta-analysis of 42 studies (55 arms) found that oral DHEA supplementation increases total testosterone by approximately 28 ng/dL, although the magnitude of increment can be higher in females compared to men [451]. More specifically, DHEA supplementation at doses ≥ 50 mg/day significantly increases testosterone levels in postmenopausal women [452]. In young men, some studies with 50–150 mg/day showed that DHEA may increase precursors (such as androstenedione) but not always testosterone directly [453].

Taken together, current evidence indicates that while certain herbal extracts such as ashwagandha and fenugreek show modest but consistent effects, the most robust and biologically supported benefits for testosterone enhancement are seen with zinc and magnesium, particularly in deficient populations. Boron and DHEA also demonstrate potential, though findings are mixed and appear context-dependent, highlighting the need for larger, well-controlled clinical trials to clarify efficacy, safety, and long-term outcomes across diverse male populations.

## 6. Conclusions

**Figure 5:**
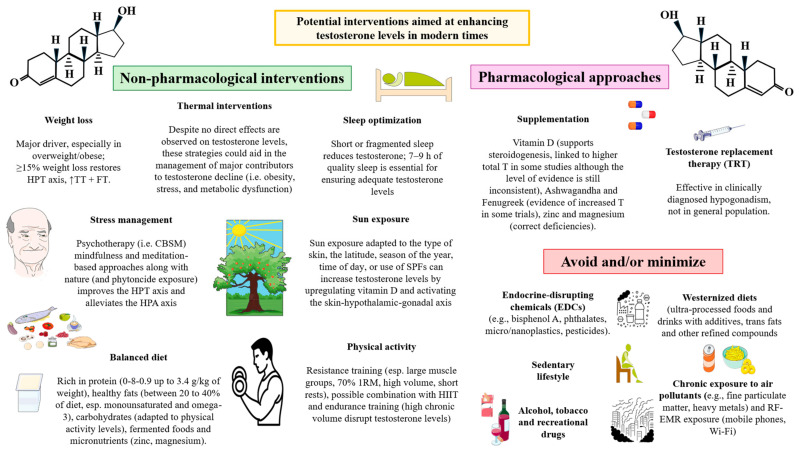
Potential interventions to restore or enhance testosterone levels in modern contexts. Non-pharmacological strategies include weight loss (≥15% body weight restores hypothalamic–pituitary–testicular [HPT] axis), resistance and combined exercise regimens, balanced diets (adequate protein, healthy fats, complex carbohydrates, and micronutrients), optimized sleep (7–9 h), stress management (psychotherapy, mindfulness, nature exposure), and sun exposure (improving vitamin D levels and activating the skin–hypothalamic–gonadal axis). Pharmacological and supplementary approaches include vitamin D, Ashwagandha, Fenugreek, zinc, and magnesium (evidence varies in consistency), as well as testosterone replacement therapy (TRT) in clinically confirmed hypogonadism. Environmental interventions involve reducing exposure to EDCs, air pollutants, RF-EMR, and limiting alcohol, tobacco, and recreational drug use.

Testosterone plays a fundamental role in male (and female) physiology, regulating reproductive capacity, muscle and bone mass, energy metabolism, mood, and overall health. The evidence for a secular, age-independent decline in testosterone levels among men is robust, with large longitudinal and cross-sectional studies demonstrating consistent reductions across generations, particularly in adolescents and young adults. This trend carries wide-ranging health implications, contributing to reduced fertility, cardiometabolic disorders, cognitive decline, psychosocial dysfunction, autoimmune susceptibility, sarcopenia, and decreased quality of life, with significant socioeconomic and public health burdens. Multiple interacting factors appear to underlie this decline, including obesity, EDCs, environmental pollutants, poor diet, physical inactivity, circadian disruption, stress, and comorbidities, with RF-EMR exposure emerging as additional contributors. While aging and genetic architecture remain key non-modifiable determinants of testosterone regulation, growing evidence supports the role of modifiable lifestyle and environmental factors (Figure 5). Interventions such as resistance training, weight loss, balanced nutrition, adequate sleep, stress management, sun exposure, and avoidance of harmful substances demonstrate consistent benefits in supporting physiological testosterone levels. Adjunctive measures including supplementation (e.g., vitamin D, ashwagandha, fenugreek), thermal therapies, and treatment of sleep-related breathing disorders may further optimize outcomes, while testosterone replacement therapy remains appropriate only for clinically hypogonadal men. Collectively, these findings highlight the urgent need to address the multifactorial causes of declining testosterone through preventive, lifestyle-centered, and evidence-based strategies, while advancing research into the long-term health consequences of this phenomenon and the most effective interventions to counteract it.

## Figures and Tables

**Table 1:** Current evidence supporting a potential secular decline in testosterone across different populations.

Study	Population and Sample Size	Age Range	Main Results
Santi et al. [164]	Global analysis of published data (1257 studies; 1,064,688 subjects)	Adults across full age spectrum	Significant annual decline of 0.56% in T levels, independent of age and assay method.
Travison et al. [12]	U.S., 1532 men, 3 waves (1987–2004)	40–70 yrs	Documented age-independent decline in testosterone; not explained by health/lifestyle (BMI, smoking). Cohort effect stronger than aging alone.
Mazur et al. [18]	U.S., longitudinal cohort	Middle-aged men	Mean total T dropped from 638 → 431 ng/dL (−33%); decline much greater than aging alone, indicating strong cohort effect.
Lokeshwar et al. [165]	U.S. adolescents and young men	15–39 yrs	Mean total T fell 605 → 451 ng/dL (−25%); significant after BMI adjustment; concerning trend in young males.
Andersson et al. [166]	Denmark, population-based	Adult men	Later-born cohorts had lower T; BMI explained part of the decline in total T, but SHBG rose independently.
Perheentupa et al. [167]	Finland, national surveys	Men 25–70+ yrs	Decline in total & free T and SHBG; in 60–69 yrs: 21.9 → 13.8 nmol/L across birth cohorts; effect remained after BMI adjustment.
Chodick et al. [13]	Israel, >100,000 men tested 2006–2019	20–70+ yrs	Highly significant age-independent drop in total T across all age groups.
Trimpou et al. [168]	Sweden, population sample	Adult men	2008 cohort had significantly lower free T than 1995 men of same age.
Walsh et al. [169]	U.S., clinical database (2002–2011)	Middle-aged to older men	Proportion with low T rose from ~35% → 47%; indirect evidence of population-level decline.
Nyante et al. [170]	U.S., NHANES men	20–75 yrs	Found no overall change in total T, but noted subgroup differences (e.g., reduced SHBG in young white men, increased free T in young Black men).

## Data Availability

No new data were created or analyzed in this study. Data sharing is not applicable to this article.

## Abbreviations

The following abbreviations are used in this manuscript:

A4	4-Androstenedione
AKT	Protein Kinase B
Ane	Androsterone
AP	Androgenic Pheromones
AR	Androgen Receptor
ARE	Androgen Response Element
BMI	Body mass index
AVP	Arginine Vasopressin
cAMP	Cyclic Adenosine Monophosphate
CNS	Central Nervous System
CREB	cAMP Response Element-Binding Protein
CYP11A1	Cytochrome P450 Family 11 Subfamily A Member 1
CYP19A1	Aromatase
CYP3A4	Cytochrome P450 3A4
DC	Dendritic Cell
DHEA	Dehydroepiandrosterone
DHT	Dihydrotestosterone
EA	Estrogenic Androgens
EDCs	Endocrine-Disrupting Chemicals
EPO	Erythropoietin
ERK	Extracellular Signal-Regulated Kinase
FSH	Follicle-Stimulating Hormone
FSH-R	Follicle-Stimulating Hormone Receptor
GH	Growth Hormone
GHRH	Growth Hormone-Releasing Hormone
GnRH	Gonadotropin-Releasing Hormone
HbA1c	Glycated Hemoglobin
HPA axis	Hypothalamic–Pituitary–Adrenal Axis
HPGn axis	Hypothalamic–Pituitary–Gonadal Axis
HPO axis	Hypothalamic–Pituitary–Ovarian Axis
HPT axis	Hypothalamic–Pituitary–Testicular Axis
HSP	Heat Shock Protein
IGF-1	Insulin-like Growth Factor 1
IL	Interleukin
KD	Ketogenic diet
KT/11-KT	11-Ketotestosterone
LH	Luteinizing Hormone
LH-R	Luteinizing Hormone Receptor
MAPK	Mitogen-Activated Protein Kinase
MD	Mediterranean diet
MDD	Major Depressive Disorder
MEK	MAPK/ERK Kinase
NF-κB	Nuclear Factor Kappa-Light-Chain-Enhancer of Activated B Cells
NK cells	Natural Killer Cells
OT	Oxytocin
PCOS	Polycystic Ovary Syndrome
REF	radiofrequency electromagnetic radiation
RORγt	RAR-Related Orphan Receptor Gamma t
SA	Serum Albumin
SHBG	Sex Hormone-Binding Globulin
STAT	Signal Transducer and Activator of Transcription
Treg	Regulatory T Cells
TNF-α	Tumor Necrosis Factor Alpha
TRT	Testosterone Replacement Therapy
UGT	UDP-Glucuronosyltransferase
VD	Vegetarian diet
